# Rice Seedling Growth Promotion by Biochar Varies With Genotypes and Application Dosages

**DOI:** 10.3389/fpls.2021.580462

**Published:** 2021-06-21

**Authors:** Minglong Liu, Zhi Lin, Xianlin Ke, Xiaorong Fan, Stephen Joseph, Sarasadat Taherymoosavi, Xiaoyu Liu, Rongjun Bian, Zakaria M. Solaiman, Lianqing Li, Genxing Pan

**Affiliations:** ^1^Institute of Resource, Ecosystem and Environment of Agriculture, Nanjing Agricultural University, Nanjing, China; ^2^School of Agriculture and Environment, UWA Institute of Agriculture, University of Western Australia, Perth, WA, Australia; ^3^School of Materials Science and Engineering, University of New South Wales, Kensington, NSW, Australia

**Keywords:** biochar, seedling growth, rice cultivars, root morphology, low molecular weight organic acids, nanoparticles, dose-dependence

## Abstract

While biochar use in agriculture is widely advocated, how the effect of biochar on plant growth varies with biochar forms and crop genotypes is poorly addressed. The role of dissolvable organic matter (DOM) in plant growth has been increasingly addressed for crop production with biochar. In this study, a hydroponic culture of rice seedling growth of two cultivars was treated with bulk mass (DOM-containing), water extract (DOM only), and extracted residue (DOM-free) of maize residue biochar, at a volumetric dosage of 0.01, 0.05, and 0.1%, respectively. On seedling root growth of the two cultivars, bulk biochar exerted a generally negative effect, while the biochar extract had a consistently positive effect across the application dosages. Differently, the extracted biochar showed a contrasting effect between genotypes. In another hydroponic culture with Wuyunjing 7 treated with biochar extract at sequential dosages, seedling growth was promoted by 95% at 0.01% dosage but by 26% at 0.1% dosage, explained with the great promotion of secondary roots rather than of primary roots. Such effects were likely explained by low molecular weight organic acids and nanoparticles contained in the biochar DOM. This study highlights the importance of biochar DOM and crop genotype when evaluating the effect of biochar on plants. The use of low dosage of biochar DOM could help farmers to adopt biochar technology as a solution for agricultural sustainability.

## Introduction

Biochar has been known generally as a carbon-rich solid residue of biomass pyrolysis in an oxygen-limited environment in a temperature range typically of 350–600°C (Boateng et al., 2015). Unlike charcoal or char ash formed under pyrolysis up to 900°C, biochar could recover most nutrients and preserve physical structure with nano-pores inside (Pan et al., 2015). The amendment of biochar to soil has widely shown positive changes in plant growth and crop productivity (Liu et al., 2013). Such effect was addressed with manipulation of plant metabolic processes (Abbas et al., 2017; Sun C. X. et al., 2017), stimulation of plant development (Rawat et al., 2019), root morphology (Xiang et al., 2017), and root exudation (Gu et al., 2016) as well as with improved nutrient availability (Biederman and Harpole, 2013; DeLuca et al., 2015). However, plant growth change with biochar was often seen as a variable with application dose (Deenik et al., 2010; Guerena et al., 2013). Particularly, soil-borne pathogen suppression with biochar was significant at a low dose (<1%, *m*/*m*) but insignificant at a higher dose of up to 3%. Although plant growth promotion in the absence of pathogen was significant regardless of dose in a pot experiment of bean grown in a biochar-added medium (Jaiswal et al., 2015). While soil resistance to soil-borne pathogens was enhanced in rice soil over the years following biochar amendment at 20 t ha^−1^ (Lu et al., 2020), Liu et al. (2013) provisioned a rational application under 40 t ha^−1^ for biochar soil amendment in agriculture, beyond which the biochar effect would turn negative on crop production.

Plant growth rates were well-known as a function of the interactions of environments, genotypes, and farm management (Hatfield and Walthall, 2015). Accordingly, crop production could be boosted either *via* breeding new cultivars or improving farm management, or both (Studnicki et al., 2016). Plant genotype could be a crucial factor in determining the biochar effect (Noguera et al., 2011; Mehari et al., 2015). In a study with rice cultivars under biochar amendment at varying doses (Noguera et al., 2011), maximum grain production could be 4-fold higher with a particular cultivar-biochar dosage combination compared with non-treated rice. In another study using six genotypes of tomato under biochar amendment at 1% (Mehari et al., 2015), *Botrytis cinereal* disease severity was unaffected for the genotype of Jasmonic acid-deficient mutant (*def1*) but reduced by 50% for the others. Thus, understanding genotype variation with biochar could be an important issue for addressing the potential of biochar to boost crop production.

Plant trait changes with biochar were often attributed to improved supply of nutrients and organic chemicals released by biochar and improved biophysical structure related to pore structure and surface functional groups of biochar (Singh et al., 2015; Gul and Whalen, 2016). As increasingly noted as biochar DOM (Bian et al., 2019), the water-soluble fraction of biochar consisted of a variety of soluble salts, colloidal minerals, and organic compounds ranging from low molecular weight (LMW) organic acids to humic-like substances (Trompowsky et al., 2005; Graber et al., 2015). The role in plant growth promotion of this small DOM pool, present generally in biochar pyrolyzed at temperatures under 550°C, was increasingly addressed (Lin et al., 2012; Lou et al., 2015; Bian et al., 2019). Compared with unwashed fresh biochar, washed biochar removed from the DOM pool reduced methane production (Lu et al., 2018) but caused less yield increase (Korai et al., 2018) in the rice paddy. Likewise, no increase in methane production was observed in the second season following biochar amendment in a rice paddy, compared with the unamended control (Zhang A. F. et al., 2012). Following soil amendment, biochar induced short-term microbial growth (Zhou et al., 2017) and increased rice growth traits only in the first crop season (Korai et al., 2018). In a low fertility soil with a vegetable crop, biochar extract (biochar DOM only) significantly increased leaf vitamin C content and decreased nitrate content compared with non-treated biochar and washed biochar (Liu X. et al., 2018). Such effects were early attributed to plant health improvement directly *via* chemical hormesis or indirectly *via* their impact on the soil microbial community (Graber et al., 2010).

Besides, the reducing capacity of biochar DOM could contribute to the reduction and solubilization of Mn and Fe oxides in soil (Graber et al., 2014), while nanosized particles in the DOM could fracture from biochar upon exposure to fluctuating redox conditions (Joseph et al., 2015). Redox-active areas, both on the biochar surface and the fracture particles, could increase electron shuttling by microbes, which in turn could increase nutrient availability (Kappler et al., 2014). Related to this, reduction of root hair density and length with a biochar extraction product containing humic-like substances was observed for *Arabidopsis* grown in P-sufficient or P-starved soil under sterile conditions (Graber et al., 2015). Moreover, nanoparticles isolated from biochar could function for plant or root development (Qu et al., 2016; Liu G. et al., 2018; Wang et al., 2019). Nanosized biochar particles were stable in the solution in the control of ionic strength, and their mobility could be increased significantly by biochar aging in soil (Wang et al., 2019). The outer and inner surfaces of biochar particles could form an organic/mineral plaque layer, which could fragment or generate nanoparticles, with the help of microbes and fine roots in soil (Hagemann et al., 2017). Hence, biochar nanoparticles could alter seed germination and plant development (Lin et al., 2009). In a soil environment, either metal-, carbon-, or Si-based nanoparticles from biochar could act in a way different from their macro-counterparts (Oleszczuk et al., 2016; Liu G. et al., 2018), and could be potentially taken up by plants *via* root cell wall and then transported to leaves (Schwab et al., 2016). Like engineered nanomaterials, biochar nanoparticles could, thus, affect the transport, retention, and availability of different contaminants and nutrients (Liu and Lal, 2015). A dose-response relationship or hermetic-like biphasic dose dependence existed for plant growth with a broad range of nanoparticles both *in vitro* and *in vivo* models (Iavicoli et al., 2014, 2018). Using silver nanoparticle (AgNP) solutions, Wang et al. (2013) found that root and stem biomass of poplars was increased at low concentrations but prohibited at higher concentrations. Similarly, carbon nanoparticles obtained from biochar were found to boost wheat (*Triticum aestivum*) plant growth in a pot experiment, which was related to the controlled and slow release of nutrients for better assimilation by plants (Saxena et al., 2014).

So far, the variation in biochar effects with genotypes and the role of organic molecules and nanoparticles in the root growth of rice have not yet been sufficiently understood. Therefore, we hypothesized that the use of different forms of biochar could bring about different effects on rice seedling growth, which could be examined with root morphology and plant assimilation activity. We further hypothesize that the biochar effect on rice seedling growth could vary with genotypes because of different biochar material responses in various forms and dosages. The objectives of this study were to determine how biochar could impact plant growth, and whether dose dependence existed for the biochar effect when nutritional and soil physical aspects of biochar amendment were neutralized in hydroponic culture. We hope to provide insights into the inherent processes potentially responsible for the biochar effect and potential opportunities to use functional types of biochar to boost seedling growth in rice agriculture.

## Materials and Methods

### Biochar Treated

Non-treated fresh biochar (NBC): maize residue biochar produced in a batch pyrolyzer at an average pyrolysis temperature of ~450°C and provided by Shanxi Gongxiao Company (Shanxi, China). The biochar was ground to pass through a 20-mesh sieve and homogenized.

Washed biochar (WBC) and biochar water-extract (BCE): the above biochar material was extracted with hot water to obtain a BCE (or biochar DOM), but the residue was left as WBC free of DOM. For this, 10 g of biochar was added to 200 ml of demineralized water and heated in a water bath at 100°C for 3 h. The mixture was then shaken with a rotatory shaker at 180 rpm at room temperature (25°C) for 24 h and subsequently vacuum-filtered through a 3-μm ceramic filter (Grade 44 Whatman, Cytiva, China). The extraction procedure was repeated twice, and the filtrate was finally pooled as a BCE stock and the residue on the filter as WBC. The liquid stock of extract was stored at 4°C before the analysis and hydroponic experiment. The analysis of biochar was performed following IBI Biochar Standards Testing Guidelines version 2.1 (IBI, 2015), while the analysis of BCE was performed following Bian et al. (2019), which was detailed in Supplementary Material. A portion of BCE was filtered through a 0.45-μm PES filter (Millipore, #SLHP033RB) and neutralized with a 30% HCl solution to achieve pH 7. The resultant solution was injected into a LC-OCD (Liquid Chromatography-Organic Carbon Detector; DOC-Labor, Germany) for organic fraction characterization (Taherymoosavi et al., 2016). Properties of the three treated biochars are provided in Table 1.

**Table 1:** Properties of non-treated fresh biochar, washed biochar, and biochar extract.

**Item**	**NBC**	**WBC**	BCE*
pH (H_2_O)	8.54	8.44	8.40
EC (ms cm^−1^)	3.34	1.79	2.34
Total N (g kg^−1^)	9.39	5.78	7.83
Total P (g kg^−1^)	3.32	1.69	12.19
Total K (g kg^−1^)	38.35	15.99	330.51
Avail-P (mg kg^−1^)	140.69 g	110	/
Avail-K (g kg^−1^)	15.18	1.97	/
Organic Carbon (%)	42.94	50.28	/
DOC (g kg^−1^)	1.72 g	0.64	70.68
CEC (cmol kg^−1^)	305.24	179.34	/
Ash (%)	36.7	36.3	/
Fe (g kg^−1^)	6.24	5.04	0.43
Ca (g kg^−1^)	33.45	35.20	33.41
Mg (g kg^−1^)	11.43	11.85	1.41
Mn (mg kg^−1^)	145.35	144.16	26.15
Cu (mg kg^−1^)	10.28	5.78	0.67
Zn (mg kg^−1^)	105.82	116.91	232.44

DOC, dissolved organic carbon; ^*^for BCE, the contents of N, P, K, Fe, Ca, and Mg, as well as DOC, are expressed in mg L^−1^; while those of Mn, Cu, and Zn are expressed in μgL^−1^.

### Experiment Setup

Two hydroponic culture experiments were carried out in this study.

#### Experiment 1: Biochar Type and Dosage

Two rice (*Oryza sativa* L. ssp. *japonica*) cultivars of Wuyunjing 7 and Nipponbare (W7 and NP, respectively, hereafter), wildly used in rice breeding, were selected for this study. Seeds of these two cultivars were sterilized in a solution of 10% (*v*/*v*) hydrogen peroxide for 30 min first and then thoroughly washed with deionized water. These seeds were germinated on a plastic supporting net (mesh of 1 mm^2^) mounted in a plastic container. After 2 weeks, six uniform seedlings of each cultivar were randomly selected to transplant to an opaque tank (Supplementary Figure 1A) containing 6 L of adjusted International Rice Research Institute (IRRI) nutrient solution (Supplementary Table 1). The nutrient solution in the tank was replaced twice per week, and pH was adjusted to 5.5 daily using 0.1M NaOH or 0.1M HCl from 7:00 to 9:00 p.m. For biochar treatment, into the tank, NBC and WBC were added in a dosage of 0, 0.05, and 0.1% (*m/v, biochar/water*) and the BCE from WBC in a dosage relevant to biochar used (that is the amount of extract from biochar used at 0.05 or 0.1%). As in hydroponic culture, the seedlings were then subject to grow in the tank with these biochar treatments. The experiment was carried out in a controlled greenhouse with four biological replicates at natural lighting with a temperature range of between 25 and 35°C in a day. Rice seedling allowed growing for 60 days.

#### Experiment 2: Biochar Water Extract Dosage vs. Nutrient Solution

The cultivar of W7 was further selected to investigate the effect of BCE on rice growth in hydroponic culture with four biological replicates. BCE was added at doses relevant to biochar at 0, 0.01, 0.05, and 0.1% (described above). Macro-nutrient balance was guaranteed by adjusting the additive volume of the IRRI nutrient stock solution (Supplementary Table 1) to ensure that the concentration of N, P, and K was consistent across the treatments. A treatment without adding the IRRI nutrient solution was treated with 0.1% BCE. In this experiment (Supplementary Figure 1B), seven rice seedlings were fixed in a plastic rigid opaque PVC sheet by sponge and grew in an opaque bucket containing 3 L of adjusted IRRI nutrient solution. The growth condition and management of rice seedlings were the same as in Experiment 1. The rice seedlings were allowed to grow for 60 days. This experiment was carried out twice.

### Leaf Photosynthesis Parameters

Leaf photosynthesis parameters were analyzed on the 60th day of seedling growth in the culture, such as transpiration rate, photosynthetic rate, intercellular CO_2_ concentration, and stomatal conductance to water vapor. These measurements were performed with an LI-6800 portable photosynthesis system (LiCor Inc., Lincoln, NE, United States) at an ambient CO_2_ level [reference CO_2_ controlled at 400 μmol mol^−1^; sample CO_2_, 386 ± 6 μmol mol^−1^ (mean ± SD)].

### Root Morphology and Plant Sample Analysis

Rice plants were harvested on the 60th day of seedling growth in the culture. Plant roots and shoots were collected, separated, and washed with distilled water. The fresh biomass of rice roots and shoots was measured.

Rice roots of selected plants were placed on a transparent tray with the necessary amount of water to allow the roots to be manipulated and located in the best position on the scanner surface to be scanned. Root samples were taken and scanned to measure total length, surface area, average diameter, and total volume using a WinRHIZO Pro LA2400 root analysis system (Regent Instruments, Inc., Quebec, QC, Canada). According to root development and diameter, the rice roots were divided into primary roots, emerged from the basal internode of stems, and secondary roots, the branch of primary roots (Yoshida, 1981). To observe the morphology of the root surface, scanning electron microscopy with energy dispersive X-ray spectroscopy (SEM-EDS) was employed as per the method described in a previous study (Joseph et al., 2013).

A portion of the plant roots or shoots was oven-dried to a constant weight at 70°C for 72 h, and the dry weight was recorded. A further portion of a plant sample was digested with H_2_SO_4_-H_2_O_2_ for nutrient content determination. In detail, the content of N was determined with indophenol blue spectrophotometric method, that of P with molybdenum blue method and that of K with flame spectrophotometry, respectively.

### Data Processing and Statistics

Following Zhang A. F. et al. (2012), biochar effect intensity (BEI) was used for highlighting the biochar effect over the other environmental factors:

(1) $$\begin{array}{r} BEI\;\left(\%\right)=\left(\frac{Q_{BC}-Q_{CK}}{Q_{CK}}\right)\times 100 \end{array}$$

where Q_BC_ and Q_CK_ are the biomass or other indicators with certain treatment and the control, respectively.

All data were expressed as mean plus/minus a standard deviation of four biological replicates and provided in Supplementary Material (SM). Differences between treatments were examined using a three-way analysis of variance (ANOVA) followed by a two-tailed Student's *t-*test. All statistical analyses of plant traits were performed using Microsoft Excel 2016 (Microsoft Corporation, Albuquerque, NM, United States) and Prism for Windows Version 7.00 (GraphPad Software, La Jolla, CA, United States).

## Results

### Changes in Rice Biomass With Different Biochar Treatments

**Figure 1:**
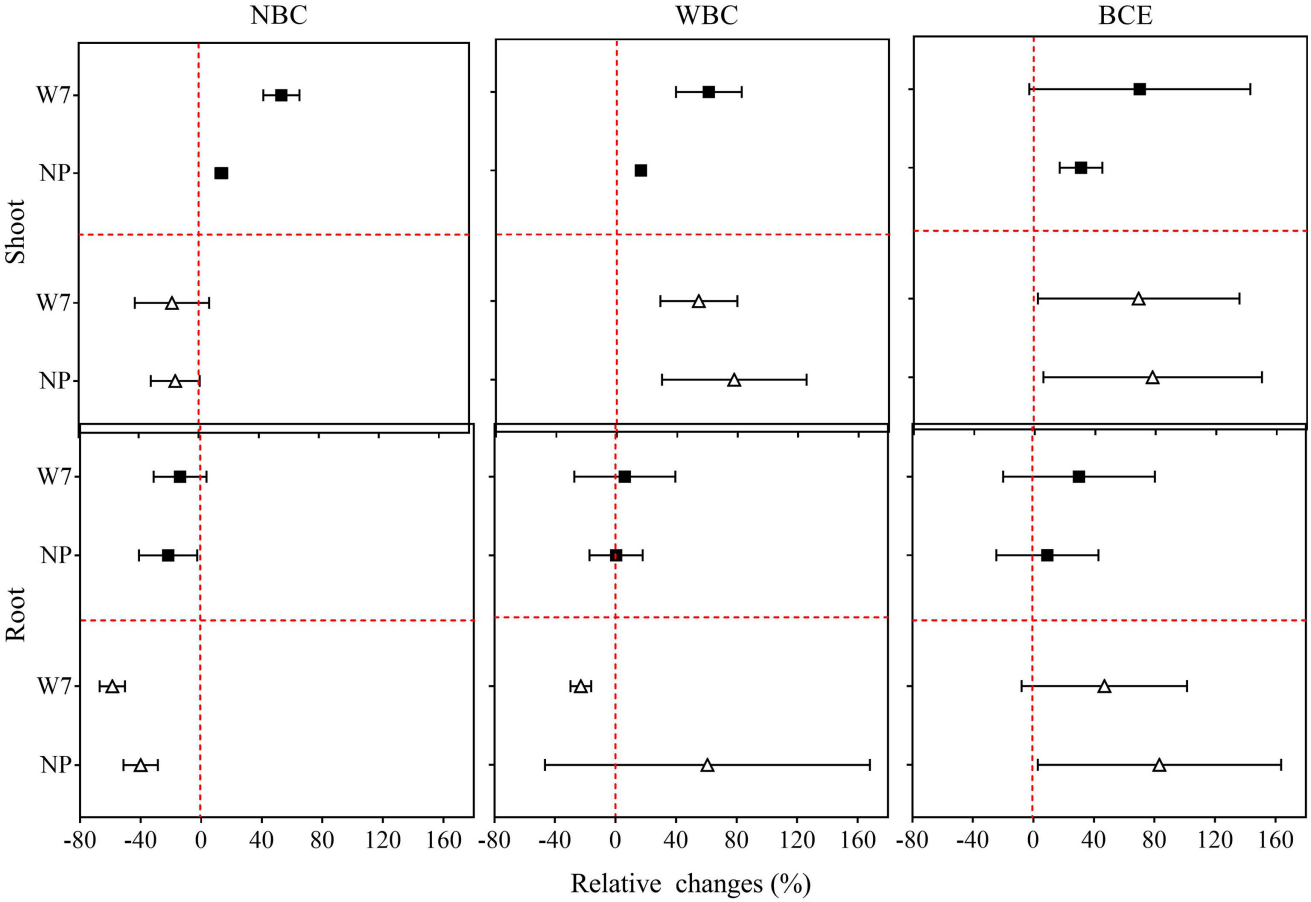
Relative changes in fresh shoots and roots biomass of Wuyunjing 7 and Nipponbare with NBC, WBC, and BCE at a dosage of 0.05% (solid square) and 0.1% (hollow triangle) in Experiment 1 (*n* = 4).

Original data of root and shoot biomass with the treatments in Experiment 1 are provided in Supplementary Table 2. As shown in Figure 1, NBC treatment at 0.1% dosage decreased fresh shoot biomass by 1 and 16%, respectively, for W7 and NP. Fresh root biomass, however, was remarkably (*P* < 0.05) decreased by 59% for W7 but unchanged for NP, compared with the control (0% biochar). With the NBC treatment at 0.05% dosage, rice shoot biomass was increased by 55% for W7 and by 15% for NP, while fresh root biomass was unchanged for W7 but decreased by 22% for NP. With WBC at 0.1%, there was a positive change in fresh shoot biomass of both cultivars (by 54% for W7 and by 78% for NP) and in fresh root biomass of NP (by 60%) but a slight negative change in fresh root biomass of W7. However, WBC at 0.05% increased fresh shoot biomass of W7 by 61% and of NP by 16%, without significant change in root biomass. Notably, BCE treatment at 0.1% increased remarkably both shoot and root biomass for both cultivars by 69 and 47% for W7, and by 78 and 83% for NP. In contrast, BCE treatment at 0.05% increased (*P* < 0.05) both the shoot and root biomass, respectively, by 69 and 30% for W7, without a significant change for NP.

### Changes in Root Morphology With Different Biochar Treatments

**Figure 2:**
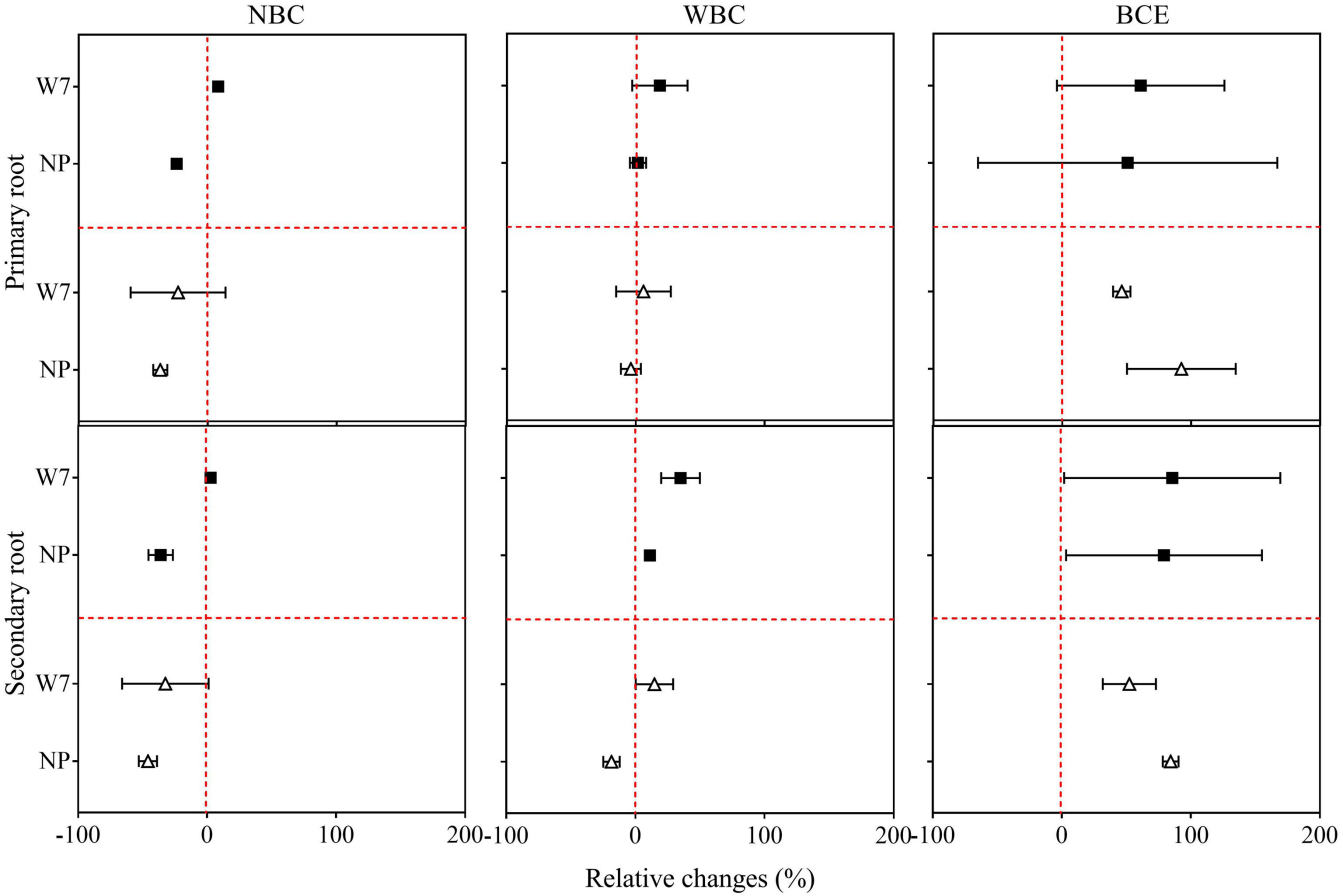
Relative changes in primary and secondary root length of Wuyunjing 7 and Nipponbare with NBC, WBC, and BCE at a dosage of 0.05% (solid square) and 0.1% (hollow triangle) in Experiment 1 (*n* = 4).

Again, original data of observation of root morphology with the treatments in Experiment 1 are listed in Supplementary Tables 3, 4. Compared with CK, decreasing trends in root growth parameters were observed for both W7 and NP with NBC treatment, while there were no significant changes with WBC treatment (Figure 2). For W7, all examined root traits were found significantly enhanced (up to 94%) with BCE at 0.05% and had a similar increase in trend (up to 66%) at 0.1% dosage treatment, compared with CK (*P* < 0.05). Whereas, for NP, root length was significantly (*P* < 0.05) increased (> 70%) with BCE both at 0.05 and 0.1%, compared with CK. The changes in other root traits of NP followed a similar trend (Supplementary Table 3). The increase over control in secondary root length (by 89%) was higher than in primary root length (by 65%) for W7, with BCE treatment at 0.05% dosage (*P* < 0.05). Such change was not observed with BCE at 0.1% dosage (Figure 2). Differences in other secondary root traits at 0.05% dosage were generally higher than at 0.1% dosage for W7, with the treatment of NBC, WBC, or BCE (Supplementary Table 4). This was also true for NP that the stimulative effect was higher at 0.05% dosage than at 0.1% dosage (*P* < 0.05). Overall, while changes in root growth were similar between primary and secondary roots across the treatments, BCE treatment exerted a significant and positive effect, but NBC or WBC had a neutral influence on root development (Supplementary Tables 3, 4).

### Rice Plant Growth and Nutrient Uptake With Biochar Water Extract Treatment

**Figure 3:**
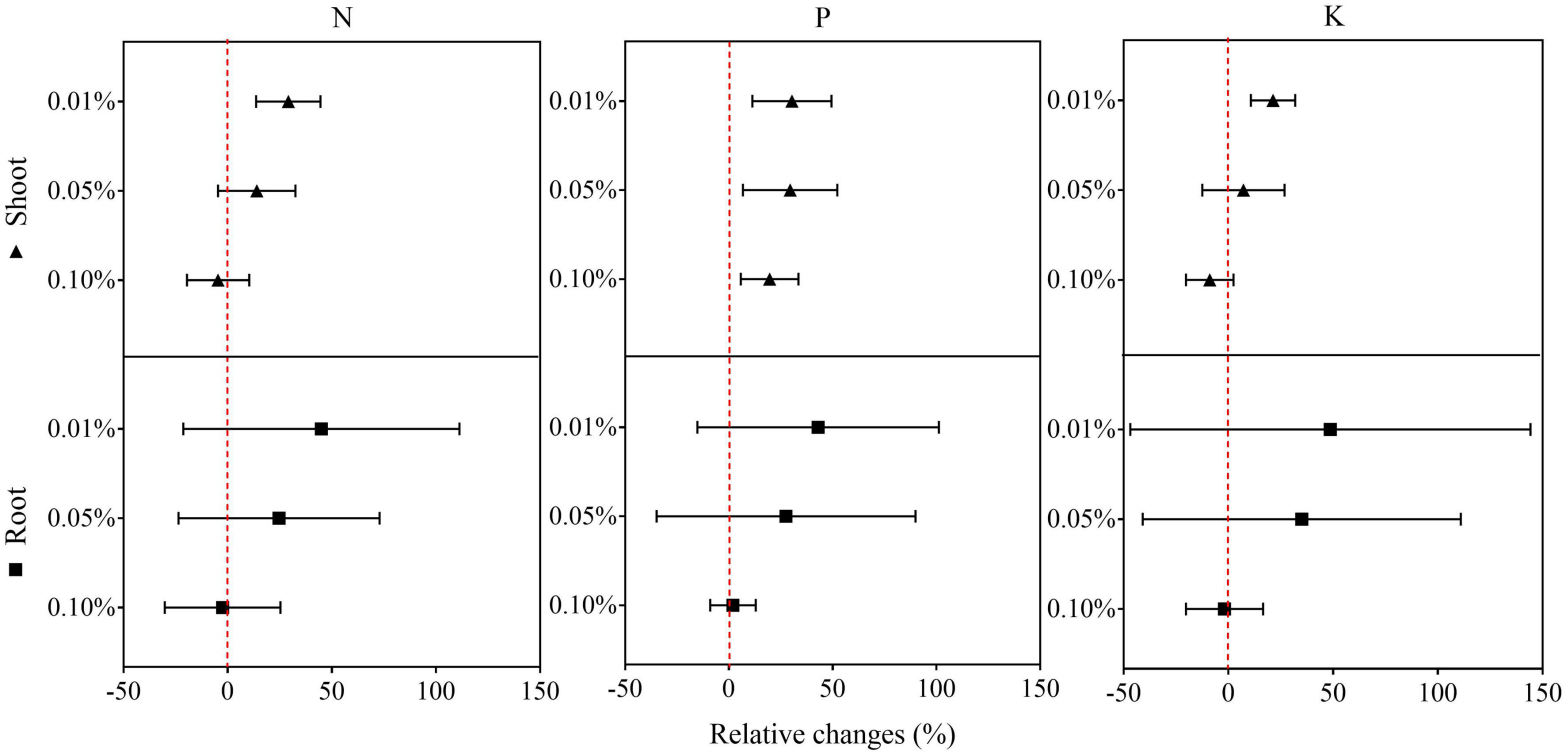
Relative changes in the N, P, and K uptake of shoots and roots across 0.01, 0.05, and 0.1% dosages in Experiment 2a (*n* = 4).

**Figure 4:**
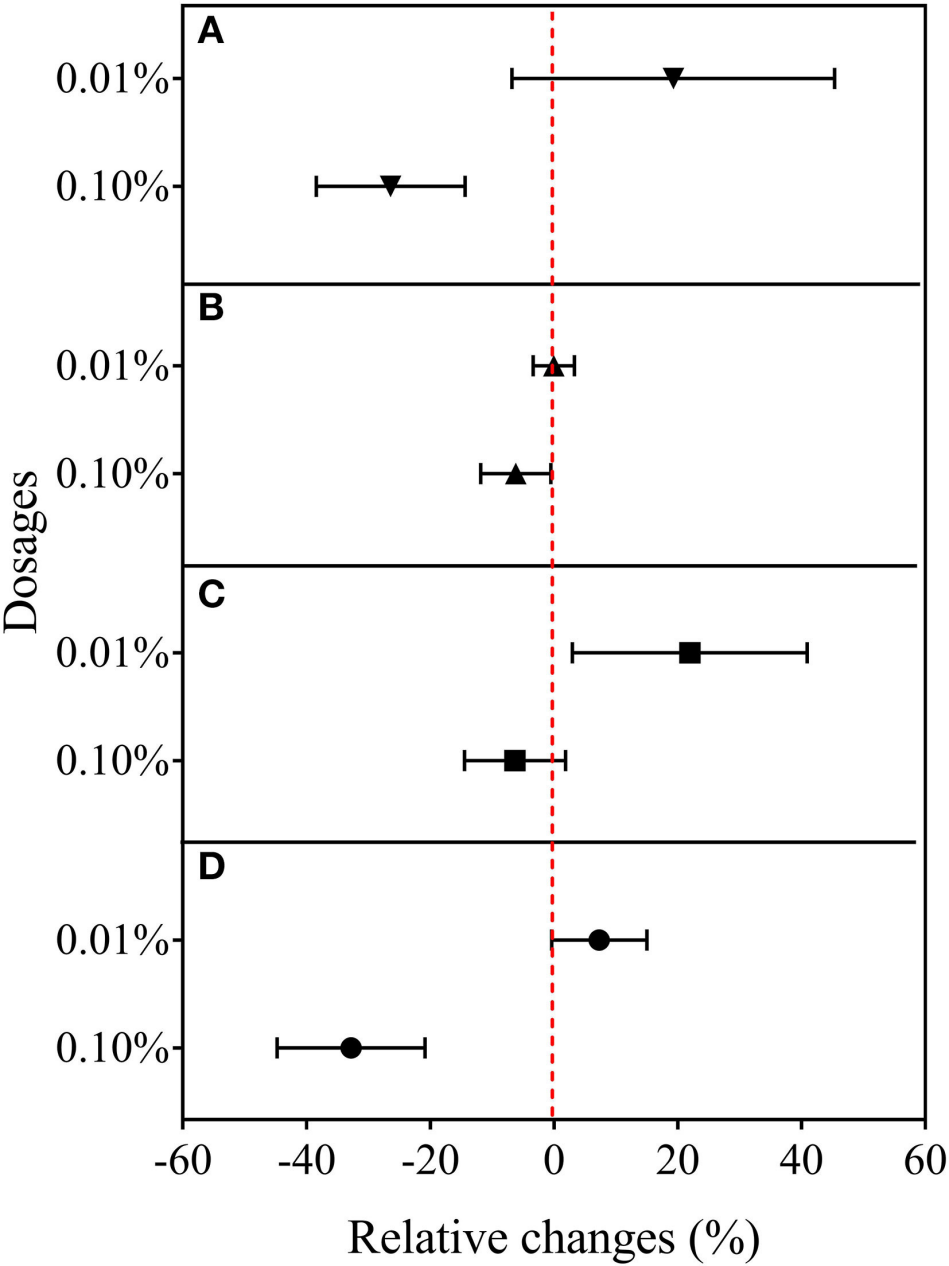
Relative changes in leaf gas exchange capacity in Experiment 2b (*n* = 4). **(A)** Leaf transpiration rate, **(B)** photosynthetic rate, **(C)** intercellular CO_2_ concentration, and **(D)** stomatal conductance to water vapor.

In the second experiment using only BCE, root dry biomass was increased by 22% at 0.01% dosage, while dry shoot biomass remained unchanged across the dosage treatments compared with CK. Plant shoot dry mass was decreased by 38.2% with 0.10% BCE without the IRRI nutrient solution (Supplementary Table 5). As shown in Figure 3, shoot nutrient contents of N, P, and K were all significantly elevated with BCE both at 0.01 and 0.05% dosages over control (*P* < 0.05). Compared with an increase in shoot nutrient content by ~30%, root contents of N, P, and K were increased by 0.8–1.08-fold with BCE at 0.01% dosage over the control. Nevertheless, shoot and root contents of both N and K were unchanged, while shoot P was significantly (*P* < 0.05) but slightly increased with BCE at 0.1% (Supplementary Table 6). Besides, the root to shoot transfer coefficient of N was greatly increased, while P and K significantly improved with BCE at 0.01% dosage over CK. Nonetheless, the nutrient transfer coefficient increased by folds over the blank with 0.1% BCE without the IRRI nutrition solution (Supplementary Table 6). For plant physiological traits (Figure 4 and Supplementary Table 7), BCE treatment significantly increased leaf area as well as leaf SPAD either at 0.01% or at 0.1% dosage (*P* < 0.05). However, BCE at 0.01% dosage did not change leaf *E, Ci*, and WUE, compared with control. In contrast, BCE at 0.1% dosage significantly (*P* < 0.05) decreased the values of *E, Ci*, and *g*_*sw*_ but increased those of WUE and WUEi.

### Rice Root Morphology With Biochar Water Extract Treatment

**Figure 5:**
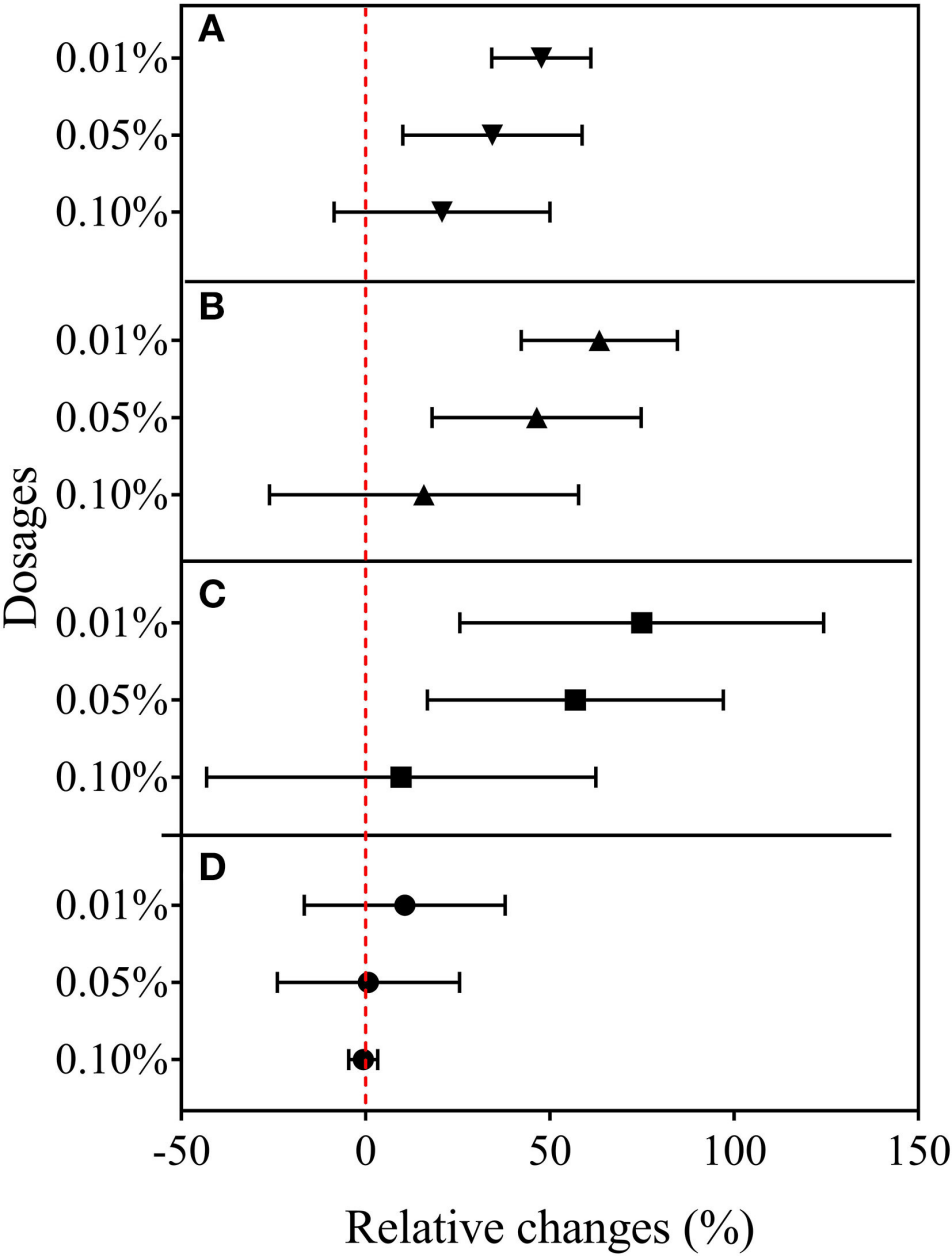
Relative changes in root morphological traits in Experiment 2a (*n* = 4). **(A)** Root length, **(B)** surface area, **(C)** volume, and **(D)** tips.

**Figure 6:**
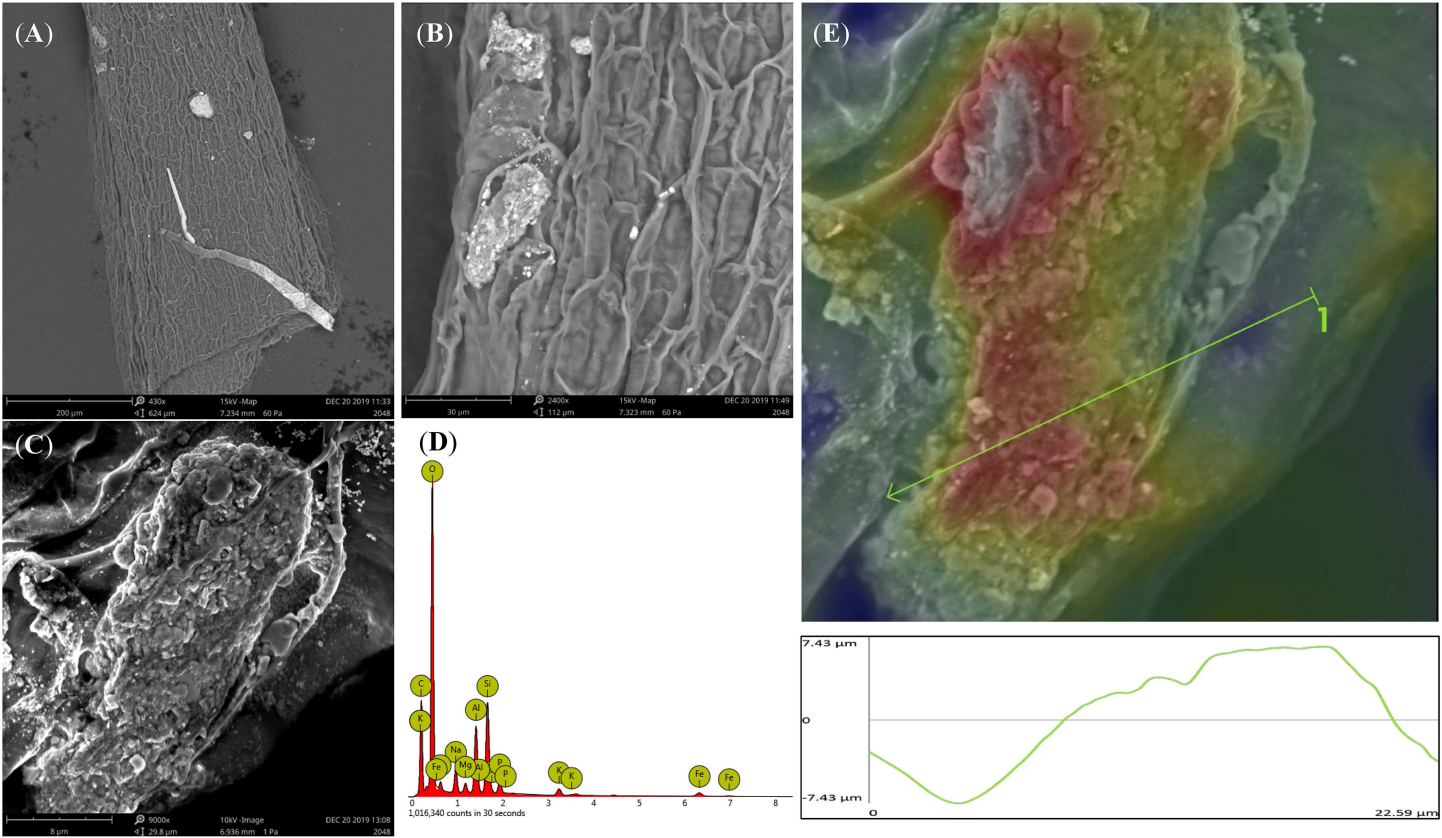
Back scattered electron images of rice root from 0.1% BCE treatment showing micron- and sub-micron-sized particles (**A**, ×400; **B**, ×2,000; **C**, ×9,000) attached to the roots. EDS image of the adhered biochar particle containing minerals and nutrients **(D)**. 3D imaging and profile measurement of the particle in **(C)**. Red color indicates top three microns of the particle **(E)**.

Rice root morphology and distribution pattern with BCE treatment in the hydroponic experiment were measured. Total root length, surface area, and volume were all increased with either 0.01 or 0.05% dosages (*P* < 0.05) but unchanged with 0.10% dosage (Figure 5). Meanwhile, total root forks and crossings were all significantly increased (by 32–54% and by 25–39%, respectively, *P* < 0.05) but tips were unchanged across the dosage treatments of BCE (Supplementary Table 8). Compared with CK, BCE treatment without the IRRI solution supply resulted in a 100–200% increase in all these root traits. However, there was a slight decrease in average root diameter (Supplementary Table 8). Changes in primary and secondary root morphology with BCE treatment generally followed a similar trend (Supplementary Table 9). While primary roots had a higher root area and volume than secondary roots, relative changes with 0.01% BCE treatment (compared with the control) were higher for secondary roots (55% on average) than for primary ones (48% on average). With the BCE applied at 0.1% without the IRRI solution, the traits of secondary roots were increased by 100–300% compared with CK, again being much higher than for primary roots. As shown in Figure 6, the interaction between the roots and the biochar was clearly observed. Imaging roots indicated that there were two types of particles that had attached to the roots. Sub-micron particles that had a crystal structure (Figure 6E) and micron-sized particles that have a similar structure and composition to the parent biochar were detected on the root (Figure 6B). Three-dimensional imaging and height profiling (Figure 6E) using backscattered electrons of the larger particle indicated that a range of smaller particles had deposited on the surface of the piece of biochar to form a micro-aggregate.

## Discussion

### Rice Growth Affected by Cultivar and Biochar Types

The results of the hydroponic experiments showed a positive effect caused by BCE addition consistently across the dosage treatments, but increasing the application rate from 0.05 to 0.1% resulted in a reduction in shoot and root biomass for both cultivars for the NBC treatment (Figure 1). This suggested dose dependence for bulk biochar addition but dose independence for biochar DOM (BCE in this study) (Table 2). Global data showed a generally negative change in crop productivity with biochar addition over 40 t ha^−1^ despite the validity of a mean increase by 10% (Liu et al., 2013). Our present study suggested the addition of biochar at low dosage (<0.1%) in rice hydroponic culture for seedling growth, regardless of biochar type, could achieve the cost-effective use of biochar, an issue regarding with biochar sue in rice agriculture (Wang et al., 2018).

**Table 2:** ANOVA table displaying *F*-values for plant shoot and root biomass, and root length, surface area, volume, and tips with three factors and all interactions across Experiment 1.

**Effect test**	**DF**	**Shoot**	**Root**	**Length**	**Surface area**	**Volume**	**Tips**
CV	1	325.72^***^	134.44^***^	228.54^***^	190.85^***^	168.37^***^	198.33^***^
BCC	2	46.00^***^	40.80^***^	260.56^***^	172.66^***^	123.99^***^	57.22^***^
BCD	2	66.56^***^	5.27^**^	37.31^***^	30.46^***^	31.10^***^	69.38^***^
CV^*^BCC	2	4.76^*^	4.70^*^	48.11^***^	38.91^***^	33.88^***^	14.38^***^
CV^*^BCD	2	22.83^***^	16.78^***^	1.48ns	2.47ns	4.03^*^	6.35^**^
BCC^*^BCD	4	34.08^***^	18.73^***^	66.01^***^	45.14^***^	34.09^***^	14.32^***^
CV^*^BCC^*^BCD	4	4.45^**^	3.52^*^	13.90^***^	12.19^***^	12.31^***^	3.85^**^

*CV, rice cultivars; BCC, biochar forms; BCD, biochar dosages; DF, degrees of freedom;*

* *P <0.05;*

** *P <0.01*;

*** *P <0.001; ns, no significant*.

Among the three forms of biochar used, bulk biochar (NBC in the study) had a generally negative effect on rice seedling growth, while BCE consistently stimulated seedling growth through divergent response to WBC for both cultivars. While washed biochar (WBC in this study) could hardly have benefits for rice agroecosystem (Korai et al., 2018), extracts from biochar did exert positive and great enhancement of plant growth and nutrient uptake (Lou et al., 2015; Bian et al., 2019). Thus, biochar boosting plant production in a hydroponic system could be mainly attributed to plant-promoting agents present in untreated biochar (Graber et al., 2015; Bian et al., 2019). The WBC solid residue after biochar washing could be considered a long-term soil physical properties improver (Enders et al., 2012), and the separated biochar DOM as a short-term plant promoter (Graber et al., 2015; Lou et al., 2015). Soil amendment of washed biochar could still be effective in immobilizing heavy metals in contaminated croplands, while the obtained extraction could be used off-site for vegetative production, raising the opportunity to valorize crop residues from metal-polluted soils (Bian et al., 2018). Of course, it is still unclear if such DOM-free biochar could bring positive and consistent effects for plants grown in soil though DOM is very limited and short-lived in a field study (Korai et al., 2018). Accordingly, the use of biochar containing a DOM pool to blend chemical nutrients had been innovated to shift a paradigm of fertilizer use in croplands to boost crop production and to minimize environmental risks (Joseph et al., 2013; Qian et al., 2014; Wen et al., 2017; Zheng et al., 2017).

For the two rice cultivars as genotype influence in this study, biochar treatment caused no difference in root biomass between the two cultivars across dosages (Figure 1). Despite this, shoot biomass promotion with biochar was found generally higher for W7 than NP at low dose (0.05%), while no difference at the dose of 0.1% across biochar forms. This change was seen as relevant to the changes in secondary root trait (Figure 2). Sun et al. (2016) showed higher root activity producing root exudates as potential nitrification inhibitor and thus higher N use efficiency for W7 than for NP. In an early study by Xu (2005), the cultivar of W7 was more resistant to soil heavy metal contamination than that of NP, which was related to high root activity and plant synthesis of endogenous hormones. Although root activity and plant metabolism were not analyzed in this study, the genotype difference in rice growth (mainly shoot) promotion with biochar at a low dose could be associated with the root activity, which could be in active response to biochar addition (Lehmann et al., 2015). While W7 had been an extensively cultivated rice cultivar in temperate regions of China (Sun et al., 2016), the use of biochar at low dosage could potentially provide an option to boost the production. Moreover, the genotype difference in shoot production suggested that screening cultivars could offer options to boost production while enhancing co-benefits in rice agriculture (Noguera et al., 2011). The two Japonica rice cultivars of W7 and NP were widely used as mother plant in rice breeding and as a model plant in rice research in China and Asian countries (Gao et al., 2011; Chang et al., 2015), and cultivar innovation by germplasm had been mostly with yield improvement by <15% (Peng et al., 2008). Thus, there could be a great potential to use a sound combination of optimal crop cultivar with biochar application in crop production improvement. Indeed, the use of biochar extract had doubled vegetative production in pot/field experiments (Lou et al., 2015; Bian et al., 2018).

### Dosage Dependence of Biochar Dissolvable Organic Matter Effect on Rice Seedling Growth

Biochar extract could stimulate rice seedling growth and root development consistently at a low dosage (<0.05% biochar equivalent) but not at a dosage of up to 0.1% in hydroponic conditions. As toxic heavy metals and organic pollutions could affect plant metabolism and tissue development (John et al., 2009; Hanano et al., 2015), one would attribute the observed promotion at low dosage to reduced plant toxicity *via* dilution of these materials present in high dosage of biochar addition. On one hand, the biochar in this study from maize residue contained an insignificant level of potentially toxic elements (Pan et al., 2015), although biochars from biosolids and animal manure could indeed have a significant pool of toxic elements (Lin et al., 2020). For example, the total content of Cu and Zn, concerned potentially heavy metals, of the biochar materials used in this study was as high as ca 200 mg kg^−1^ (Table 1), and the solution concentration of them from added biochar was as low as <0.3 μM, much lower than the nutrient solution provided (Supplementary Table 1). On the other, toxic organic pollutant molecules were not detected in the biochar extract (Supplementary Table 11). However, the fact that nutrient (NPK) uptake and root to shoot transfer were generally enhanced with BCE treatment, generally in line with the trend in seedling growth and biomass buildup, suggested plant metabolic activity indeed improved with biochar DOM at a low dose. Dilute spraying (>300 times) of biochar extract had been often recognized as a bio-promoter stimulating root growth, biomass accumulation, and nutrient uptake and nutrition quality buildup in a soil-plant system (Lou et al., 2015; Bian et al., 2019). Such promotion of the extract from crop residue-derived biochar was attributed to the presence of LMW organic acids and biopolymers (Bian et al., 2019), which could manipulate growth-related gene auxin binding protein and its encoded protein (E et al., 2019). In a study (Sun J. et al., 2017), maize seed germination was promoted by dilution of acid extract of maize biochar, but dilution of wheat biochar prohibited it. This was attributed to humic-like supramolecular structures capable of adhering on target surfaces and slowly delivering hormone-like molecules with the maize biochar extract. Nevertheless, Smith et al. (2012) noted an inhibition on the growth of some typical microbial communities in aquatic systems by wood biochar-derived water-soluble substances in a bioassay study. They could attribute this to the presence of neutral (center-isolated) and positive (cathode-isolated) electro-static compounds. Instead, the use of anode-isolated substances indeed recovered fully the growth formerly prohibited in the treated system. A recent study has shown that diluted spraying of a biochar extract rich in LMW organic acids (LMW-OA) and mineral nutrient contents exerted great promotion on vegetable leaf biomass and nutrition quality, and that the gene expression of enzyme activities had been improved for nutrient assimilation and protein synthesis in the tested plant (Bian et al., 2019). Yet, how the concentration mediated the beneficial role of biochar extract is still unknown in this study.

Plant physiological responses to biochar could be the key to the understanding of the biochar effect (Kammann and Graber, 2015), which had been explored in some recent studies (Viger et al., 2014; Graber et al., 2015; E et al., 2019). In Experiment 2, BCE treatment exerted a significant increase in leaf area both at 0.01 and 0.1% dosages, but in leaf SPAD value and net photosynthetic rate only at 0.01% dosage (Supplementary Table 7). Coincidently, higher biomass accumulation was observed under BCE only at 0.01% dosage. This was similar to the findings of Xu et al. (2015) and Abid et al. (2017) that dilute biochar extract led to an increase in leaf chlorophyll content, leaf area and turn in net photosynthetic rate of plants in soil condition. Such promotion of leaf activity could be partly explained by the increased level of leaf N, which was considered a critical determinant of the photosynthetic capacity of plant leaves (Ohsumi et al., 2007). Moreover, biochar extract had a negative effect on rice leaf gas exchange capacity (*E, Ci*, and *g*_*sw*_) at high dosage although neutral or positive at low dosage (0.01%). Solaiman et al. (2010) reported an increase in leaf conductance and internal CO_2_ concentration by biochar soil amendment at a rate lower than 6 t ha^−1^. Similarly, Batool et al. (2015) reported an increase with biochar amendment in leaf stomatal conductance and gas exchange rate under water stress conditions at an application rate of under 1% over the higher rate of 3%; whereas increased water use efficiency was reported in water-stressed rice plants grown in solution culture with multiple genotypes (Cabuslay et al., 2002). As shown in Supplementary Table 7, a marked increase in WUE (by 39.3%) and in WUEi (by 26.5%) was observed with BCE at 0.1% dosage but not at 0.01% dosage, indicating stress could exist with BCE at a higher dosage of 0.1%.

**Figure 7:**
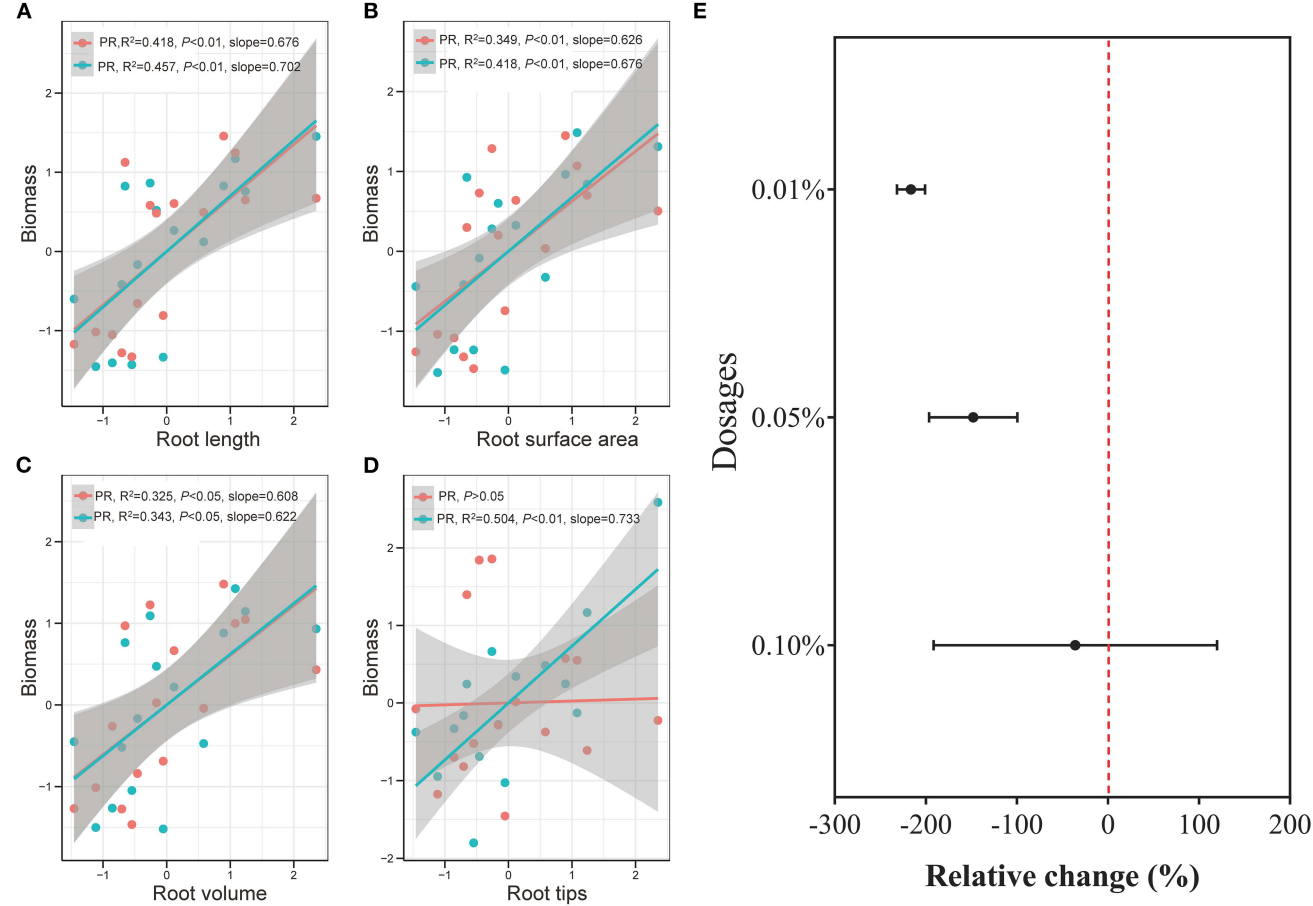
Correlation between rice biomass and rice root length (**A**, *n* = 16), surface area (**B**, *n* = 16), volume (**C**, *n* = 16), and tips (**D**, *n* = 16), and between BCE dosages and rice growth traits (**E**, *n* = 42) in Experiment 2a. Data were normalized by the Z-score scaling method (detailed in the Supplementary Material) and the shaded bands with gray color show 95% pointwise confidence intervals. PR stands for primary roots, and SR stands for secondary roots.

Overall, plant traits, such as plant biomass, plant nutrient uptake, leaf growth and activity, and root development showed a generally consistent positive increase with BCE at 0.01% dosage but divergent changes at 0.1% dosage. This indicated a dose-dependent function (Figure 7E) with biochar extract addition for rice seedling growth, which was noted early by Moffett et al. (2015). In a study on pathogen-infected bean and cucumber in a potting mixture amended with biochar at different rates (Jaiswal et al., 2015), disease suppression was maximal with biochar from pepper waste at 0.5% and from eucalyptus wood chips at 1%. Such dose-dependent effect was also reported with grass growth in biochar-amended soils at rates from 0.2 to 5% (Gale and Thomas, 2019). Thereby, the optimal dosage varied with biochar types and plant types/genotypes, but most plant eco-physiological traits, such as photosynthetic capacity, chlorophyll content, and stomatal conductance, showed peak positive responses to biochar at a dosage of ~2–3% (Gale and Thomas, 2019). With optimum growth at an intermediate biochar concentration, this pattern was referred to as a bell-shaped biochar dose/plant growth relationship in biochar/cropping systems (Jaiswal et al., 2015; Gale and Thomas, 2019). While the high rate of biochar application had often been in debt for cost-effectiveness (Clare et al., 2015), the one-time medium rate of biochar amendment (20 t ha^−1^, ca. 1% to topsoil) in a Chinese rice paddy had brought out a 6-year long net gain of crop productivity and economic return (Wang et al., 2018). Therefore, the technology for value-added biochar use should be urged to make biochar production and application viable (Clare et al., 2015; Pan et al., 2015), such as biochar-based fertilizer (Joseph et al., 2013) or valued use of separated biochar DOM (Bian et al., 2019). With this, a low dosage of DOM could offer an opportunity to cut down biochar application but increase plant production in aquatic systems.

### Secondary Root as a Key Driver for Biochar Effect

The biochar effect on crops has been more recently addressed with root development or soil-root-biochar interfaces (Prendergast-Miller et al., 2014; Lehmann et al., 2015; Xiang et al., 2017). Biochar extract improved root development apart from the enhancement of root uptake of N, P, and K nutrient at a low dosage. This coincided with the general understanding that biochar significantly and positively affected most, if not all, of root parameters and increased root P but not N concentration (Xiang et al., 2017). As shown in the correlation between root morphology and rice biomass, secondary roots had higher variability than primary roots, especially for root tips (Figures 7A–D). Moreover, 0.01% BCE dosage had the highest stimulating effect among the treatments with up to 0.1% dosage on secondary root length (54%) and surface area (55%), which was mainly correlated with water and nutrient absorption. The observation could suggest secondary rather than primary root access to the dilute soluble organic matter provided by the biochar DOM. This was not in agreement with the finding by Graber et al. (2015) who reported a significant reduction with extracted products from pepper residue biochar on root hair density and length of *Arabidopsis* treated with P supply level. Unlike in their study with the plants and culture sterilized, potential interaction of microorganisms-root-biochar could occur (Lehmann et al., 2015), resulting in improved secondary or root hairs growing in the hydroponic culture in this study.

A vigorous root system was responsible for the development of healthy plants and, in turn, higher yields. Root acquisition of nutrient and water relied rather on root length and surface area than the root mass, since high root length density was linked to a short travel distance of water and solutes, especially in soil conditions (Eissenstat, 1992; Fageria, 2012). Rice root length and root surface area were both highest, with a great number of root tips, with biochar extract at low dosage with adequate nutrition, making roots competitive enough to acquire the limited resource under stress conditions for root tips could act as local sensors to recognize objects and alter root architecture for rice grown in a stressed transparent gel system (Fang et al., 2013). Consequently, root architecture was modified by the biochar DOM at a low dosage for optimized water (Supplementary Table 10) and nutrient acquisition (Figure 2), being consistent with ideotype root phene (Lynch, 2013; York et al., 2013). Moreover, a vigorous root system with high biomass production and significant NPK uptake of rice seedling in the early stage could benefit rice reproductive growth in a later stage (Wang et al., 2016). Being mainly responsible for the absorptive surface to allow nutrient and moisture uptake for plant growth (Eissenstat, 1992; Debi et al., 2003; Comas et al., 2013), secondary root development together with increased root length promotion could be considered to drive the very marked enhancement of biomass production with dilute biochar extract during rice seedling growing.

### Hormone-Like Molecules and Nanoparticles Functioning for Root Growth

BCE as the separated DOM pool of the biochar used could contain soluble carbon groups, such as LMW acids and neutral molecules, and humics and their oxidation products (building blocks) (Table 3). As identified by the GC-MS analysis, various organic compounds existed in BCE, as the liquid extract from biochar (Supplementary Table 11). Some of these compounds, such as lactic and amino acids, have been already known as direct bio-stimulator of plant growth (Yoshikawa et al., 1993; Popko et al., 2018). The observed increase in nutrient uptake and the large increase in biomass were related to the relatively high concentration of these water-soluble organic molecules in this study. For example, the DOM from Sphagnum peat was concerned as hormone-like molecules by humic-like macromolecules (Schmidt et al., 2007). It could directly interact with rice plant roots and against oxidative stress (García et al., 2012). Water-soluble humic substances could be linked to the protein expression of soybean *via* the mediation of energy production, nucleic acid metabolism, carbon metabolism, and some transmembrane transportation in the plant body (Gao et al., 2015). Apart from these humic-like substances, LMW in water-soluble fractions could affect the functionality of ion transporters operating in the plasma membrane of root cells, acting both at the transcriptional and post-transcriptional levels (Zanin et al., 2019). In addition, organic compounds of amino acid, sugars, and organic acids could be directly taken up by plants (Nasholm et al., 2009), N-containing and N-free LMW organic substances by the maize plant (Biernath et al., 2008). Yet, how rice seedlings responded to the molecules from the added biochar extract in the hydroponic condition was not clear.

**Table 3:** LC-OCD quantitative analysis of abundance (mgg^−1^) of organics in biochar extract (DOM).

**DOC**	**TDN**	**CDOC**	**CDOC composition**				
			**Bio-polymers**	**Humic**	**Building blocks**	**LMW neutrals**	**LMW acids**
1.33	0.11	1.0	0.01	0.82	0.11	0.06	0.003

*DOC, dissolved organic carbon; TDN, total dissolved nitrogen; CDOC, chromate-graphable organic carbon; LMW, low molecular weight*.

Nanoparticles isolated from biochar could also contribute to the biochar effect on plant growth (Oleszczuk et al., 2016; Chen et al., 2017; Yue et al., 2019) as nanoparticles up-taken by plant involved in many processes, such as seed germination, root growth, and photosynthesis (Ma et al., 2010). As shown in Figure 4, biochar nanoparticles adhered to the rice root surfaces were clearly observed, of which an average particle size was 180 ± 17.83 nm and Zeta potential was −38.5 ± 6.68 mV. Some beneficial effects on plant growth by the compounds isolated from a humic substance or compost were concerned with nanoparticles occurring in these materials (Schmidt et al., 2007; Monda et al., 2017). The presence of nanoparticles on the root surface could modify the surface chemistry of the roots and consequently affect the uptake of nutrients into the plant roots (Sooyeon et al., 2012; Mirzajani et al., 2013; Ruttkay-Nedecky et al., 2017). Water-soluble carbon nanoparticles isolated from rice biochar could significantly promote the growth of wheat in soil, for the slow release of cationic and anionic nutrients preserved in their pore system over time (Saxena et al., 2014). Compared with micro-particles of biochar, these biochar nanoparticles present in the liquid phase had a larger surface area, smaller pore size, and higher negative zeta potential (Oleszczuk et al., 2016). Such nano-biochars could be formed during lignocellulose biomass degradation (Zhang M. et al., 2012; Yao et al., 2013; Oleszczuk et al., 2016; Wang et al., 2019). To readily enter the plant cell, biochar nanoparticles could be bound to organic compounds or coated with organic compounds (Cifuentes et al., 2010; Zhao et al., 2012). In this study, a particle of micro-aggregate was seen formed on the surface of a biochar piece adhered to the root (Figure 6). Overall, stimulation effects of biochar on plant growth could result from a combination of nutrients, organic compounds, and nanoparticles directly and/or indirectly (Yakhin et al., 2016; Iavicoli et al., 2018) through a study showed no particular or additive effects by nanoparticles in nano-fertilizers compared to conventional organic/mineral compound fertilizers (Kah et al., 2018).

## Conclusion

In a biochar-hydroponic experiment, rice seedling growth varied with cultivars, biochar forms, and dosages, while they had a very significant interactive role in rice root traits and biomass production. Dose dependence with BCE addition for rice growth and substances existing in biochar DOM could act as a bio-stimulator at a low dosage under hydroponic conditions. Secondary roots were deemed the key driver of the biochar plant effect because of their high response and sensitivity at various BCE dosages, closely correlated to water and nutrient uptake. These findings highlighted the importance of biochar in stimulating lateral root system development to enhance the uptake of nutrients and water to boost biomass production. LMW organic compounds as hormones-like molecules with possible nanoparticles derived from biochar could be considered a potential acting agent for plant growth. Of course, further pot experiments or field studies are deserved to explore the intrinsic process for a sound understanding of a small but effective use of biochar in the soil-plant system.

## Data Availability Statement

The original contributions presented in the study are included in the article/Supplementary Material, further inquiries can be directed to the corresponding author/s.

## Author Contributions

ML performed experiment and analysis as well as drafting of the manuscript. ZL and XL participated in the experiment. XF provided advice on rice cultivars and data interpretation. SJ and ST performed microscopic analysis. RB, ZS, SJ, and LL participated in data analysis and manuscript preparation. GP supervised the study and completed manuscript editing. All authors contributed to the article and approved the submitted version.

## Conflict of Interest

The authors declare that the research was conducted in the absence of any commercial or financial relationships that could be construed as a potential conflict of interest.

## Supplementary Material

Supplementary Figure 1 Schematic diagram of the hydroponic Experiment 1 and Experiment 2.

Supplementary Figure 2 Morphological phenotype of Wuyunjing 7 in Experiment 1.

Supplementary Figure 3 Morphological phenotype of Nipponbare in Experiment 1.

Supplementary Figure 4 Morphological phenotype of Wuyunjing 7 in Experiment 2a.

Supplementary Figure 5 Morphological phenotype of Wuyunjing 7 in Experiment 2b.

Supplementary Table 1 Composition of adjusted IRRI nutrient solution.

Supplementary Table 2 Fresh shoot and root biomass of Wuyunjing 7 in Experiment 1.

Supplementary Table 3 Rice root morphology of Wuyunjing 7 in Experiment 1.

Supplementary Table 4 Rice root distribution parameters of Wuyunjing 7 in Experiment 1.

Supplementary Table 5 Dry shoot and root biomass of Wuyunjing 7 in Experiment 2a.

Supplementary Table 6 Rice nutrients content and transfer coefficient of Wuyunjing 7 in Experiment 2a.

Supplementary Table 7 Rice leaf photosynthetic traits of Wuyunjing 7 in Experiment 2b.

Supplementary Table 8 Rice root morphology of Wuyunjing 7 in Experiment 2a.

Supplementary Table 9 Rice root distribution of Wuyunjing 7 in Experiment 2a.

Supplementary Table 10 Shoot and root water content of Wuyunjing 7 in Experiment 2.

Supplementary Table 11 Putative identifications with gas chromatograph-mass spectrometer (GC-MS) of compounds in hot water extract from maize residue biochar.

Supplementary Table 12 List of rice growth traits used to calculate relative changes by the Z-score normalization method in Experiment 2a.
