# Supplementary material for: Rice Seedling Growth Promotion by Biochar Varies With Genotypes and Application Dosages
Source: Front Plant Sci. 2021 Jun 21;12:580462. doi: 10.3389/fpls.2021.580462 (PMC8256797; doi:10.3389/fpls.2021.580462)
Supplement: Supplementary Figure 1 — Schematic diagram of the hydroponic Experiment 1 and Experiment 2. [file Data_Sheet_1.docx]

Supplementary Material

# Materials and methods

- 1. **Basic property of NBC, WBC and BCE**

The pH and Electrical Conductivity (EC) analysis procedures of NBC and WBC were using a dilution of 1:20 biochar: deionized H_2_O (*w*/*v*) and equilibration on a rotatory shaker at 180rpm at room temperature (25ºC) for 90 minutes. The pH and EC of NBC, WBC and BCE were measured using a compound glass electrode (SevenEasy Mettler Toledo, China). The total nitrogen was determined by Kjeldahl procedures. Organic carbon of NBC and WBC was determined by dichromate redox methods. The dissolved organic carbon (DOC) analysis procedures of NBC and WBC was extracted using a dilution of 1:20 biochar: ultrapure H_2_O (*w*/*v*) and heated in a water bath at 100ºC for 3 hours, then the mixture was then shaken on a rotatory shaker at 180rpm at room temperature (25ºC) for 24 hours and filter samples using a syringe and 0.45μm luer fit syringe filter. DOC of NBC, WBC and BCE was determined with an automated TOC Analyzer (Multi N/C 3100, Analytic Jena AG, Germany). The cation-exchange capacity (CEC) of NBC and WBC was measured by the ammonium acetate method at pH 7.0. The ash content of NBC and WBC was determined using a muffle furnace at 750ºC for 6 hours, then cooled in a desiccator for 1 hours and weighed. The available phosphorous was measured using 2% formic acid followed by spectrophotometry. The available potassium was measured by 1 M NH_4_OAc at pH 7.0 with flame spectrophotometry. In addition, samples were digested with HNO_3_-HClO_4_ (4:1, *v*:*v*) and total macronutrients (P, K) and total micronutrients (Fe, Mn, Cu, Zn, Ca, Mg) was determined with a graphic furnace Atomic Adsorption Spectrophotometer (A3, Persee Analytical Instruments, China). For average particle size and Zeta potential, 0.5 g of fine biochar sample was added to 50 mL double distilled water and then sonicated for 30 min. After filtration through 0.45-μm filters, the Zeta potential of colloidal biochar was determined without pH adjustment.

# Supplementary Figures and Tables

## Supplementary Figures


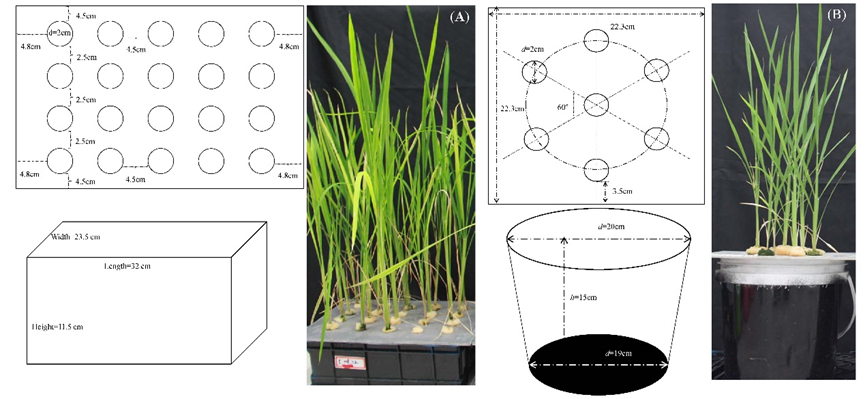


**Supplementary Figure 1.** Schematic diagram of the hydroponic Experiment 1 (**A**) and Experiment 2 (**B**), with the containers covered by black paint for rice root growth in dark. The container of Experiment 1 (**A**) was made of plastic rigid opaque PVC sheet with a thickness of 0.6 cm and a turnover box using polypropylene material; The container of Experiment 2 (**B**) of plastic rigid opaque PVC sheet with a thickness of 0.3cm and a bucket using polypropylene material, respectively.


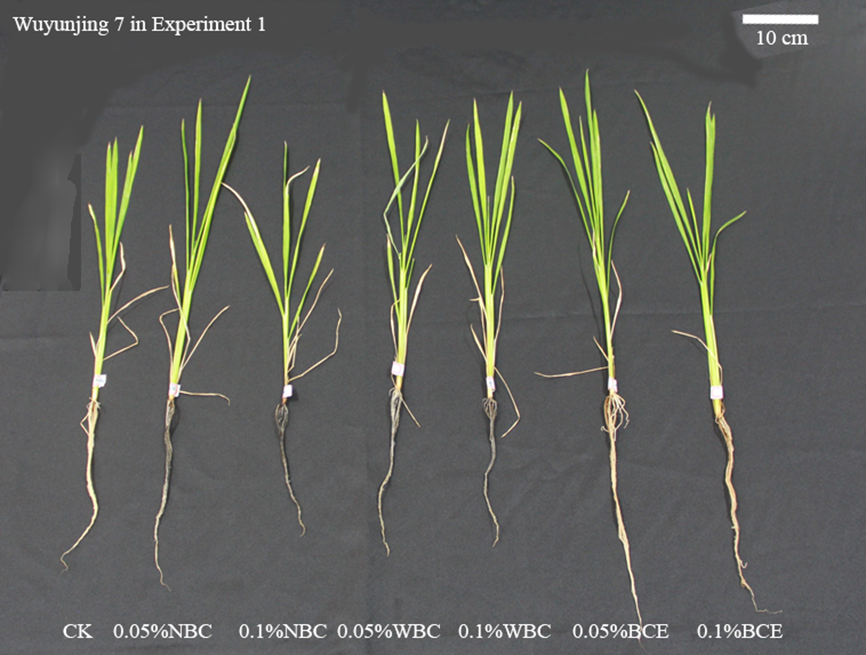


**Supplementary Figure 2.** Morphological phenotypes of Wuyunjing 7 under NBC, WBC and BCE at a dosage of 0.05% and 0.1% respectively in Experiment 1.


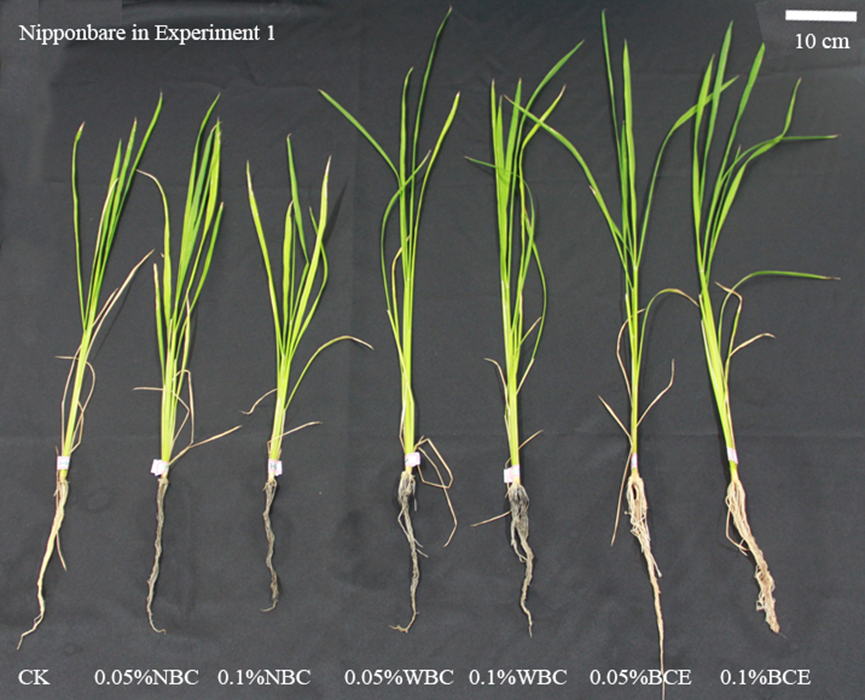


**Supplementary Figure 3.** Morphological phenotypes of Nipponbare under NBC, WBC and BCE at a dosage of 0.05% and 0.1% respectively in Experiment 1.


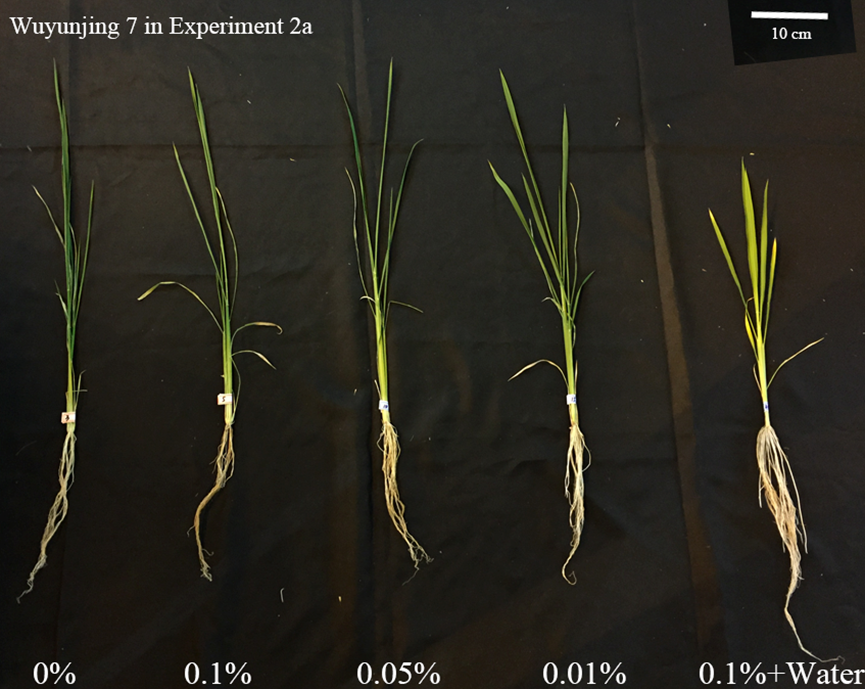


**Supplementary Figure 4.** Morphological phenotypes of Wuyunjing 7 under 0%, 0.01%, 0.05% and 0.1% BCE and 0.1% BCE alone treatments in Experiment 2a.


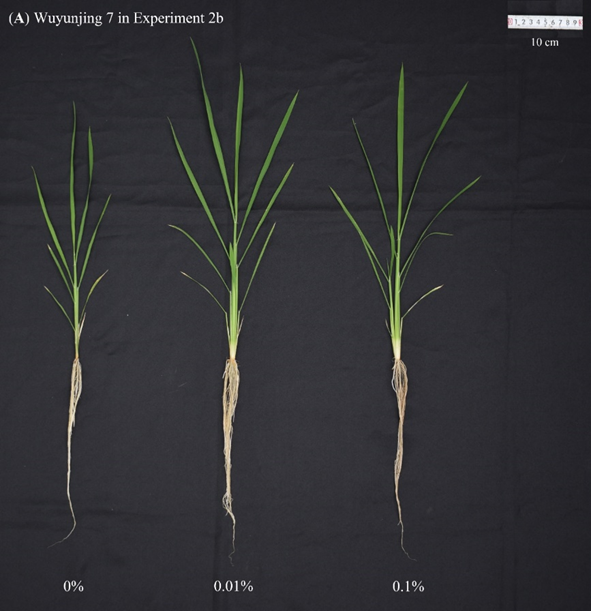

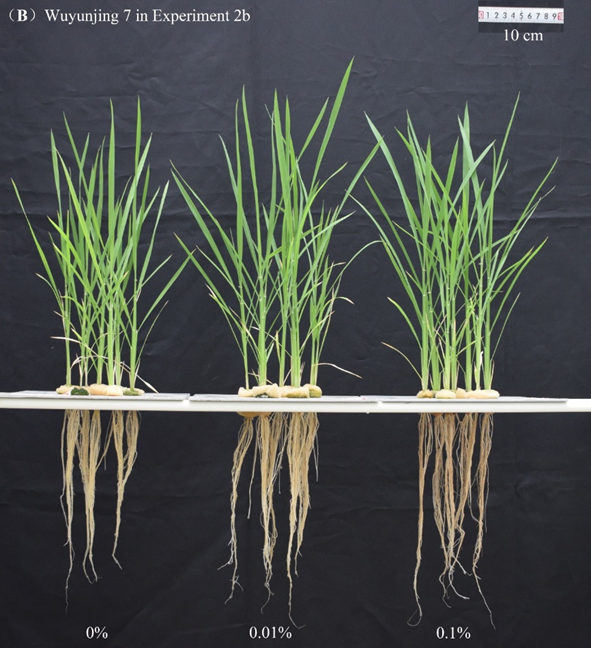


**Supplementary Figure 5.** Morphological phenotypes of Wuyunjing 7 under 0%, 0.01% and 0.1% BCE treatments in Experiment 2b. A, single rice plant; B, a pot of rice plants.

## Supplementary Tables

**Supplementary Table 1.** Composition of adjusted IRRI nutrient solution for rice seedlings in hydroponic culture

| Compound | Concentration |
| --- | --- |
| NH_4_NO_3_, | 1.25 mM |
| KH_2_PO_4_ | 0.30 mM |
| K_2_SO_4_ | 0.35 mM |
| CaCl_2_·2H_2_O | 1.0 mM |
| MgSO_4_·7H_2_O | 1.0 mM |
| Na_2_SiO_3_·9H_2_O | 0.5 mM |
| MnCl_2_·4H_2_O | 9.0 μM |
| Na_2_MoO_4_·2H_2_O | 0.39 μM |
| H_3_BO_3_ | 20 μM |
| ZnSO_4_·7H_2_O | 0.77 μM |
| CuSO_4_·5H_2_O | 0.32 μM |
| NaFeEDTA | 20 μM |

**Supplementary Table 2.** Fresh shoots and roots biomass (g plant^-1^) of rice seedlings of the two cultivars in Experiment 1. Different letters in a single column indicate significant differences between treatments at *P*<0.05 (n=4).

| Treat-ment | Biochar  dose | Shoot | | Root | |
| --- | --- | --- | --- | --- | --- |
|  |  | W7 | NP | W7 | NP |
| CK | 0% | 1.29±0.15b | 2.22±0.22cd | 0.45±0.09bc | 0.68±0.11b |
| NBC | 0.05% | 1.99±0.06a | 2.56±0.04bc | 0.39±0.04bc | 0.53±0.07bc |
|  | 0.10% | 1.06±0.17b | 1.88±0.2d | 0.19±0.03d | 0.41±0.05c |
| WBC | 0.05% | 2.07±0.11a | 2.58±0.03bc | 0.48±0.07b | 0.68±0.06b |
|  | 0.10% | 1.98±0.13a | 3.95±0.38a | 0.35±0.02c | 1.09±0.28a |
| BCE | 0.05% | 2.18±0.35a | 2.90±0.14b | 0.58±0.1a | 0.74±0.11b |
|  | 0.10% | 2.17±0.32a | 3.96±0.58a | 0.66±0.1a | 1.24±0.19a |

**Supplementary Table 3.** Changes in root morphology parameters of projected area, surface area, average diameter, volume, tips, forks and crossings of W7 and NP under different biochar forms in Experiment 1. Different letters in a single column indicate significant differences between treatments at *P*<0.05 (n=4).

| Cultivar | Treatment | Length  (cm) | Projected area  (cm^2^) | Surface area  (cm^2^) | Average diameter  (mm) | Volume  (cm^3^) | Tips | Forks | Crossings |
| --- | --- | --- | --- | --- | --- | --- | --- | --- | --- |
| W7 | CK | 914±13d | 24.84±0.97ef | 78.03±3.06ef | 0.272±0.007d | 0.530±0.034d | 4134±184d | 6891±235c | 1141±51bc |
|  | 0.05NBC | 954±5d | 26.55±0.36de | 83.40±1.12de | 0.278±0.005cd | 0.580±0.018d | 5490±101c | 7243±64c | 997±19c |
|  | 0.10NBC | 646±184e | 19.83±5.44f | 62.29±17.09f | 0.308±0.003a | 0.478±0.126d | 4325±965d | 5306±1517d | 652±196d |
|  | 0.05WBC | 1188±67bc | 33.67±0.72c | 105.76±2.26c | 0.284±0.010c | 0.750±0.011c | 7211±221a | 8970±625b | 1266±212b |
|  | 0.10WBC | 1027±72cd | 30.36±2.26cd | 95.38±7.10cd | 0.295±0.001b | 0.705±0.056c | 6100±584bc | 8041±189bc | 1095±44bc |
|  | 0.05BCE | 1627±252a | 45.86±6.87a | 144.06±21.59a | 0.282±0.001c | 1.016±0.147a | 7031±1328ab | 11320±1961a | 1855±280a |
|  | 0.10BCE | 1371±60b | 39.18±2.48b | 123.10±7.79b | 0.285±0.006c | 0.880±0.073b | 5588±268c | 9021±362b | 1257±90b |
| NP | CK | 1363±99bc | 38.56±3.68c | 121.14±11.57c | 0.282±0.006cd | 0.858±0.101cd | 6646±577d | 11174±1429b | 1861±216bc |
|  | 0.05NBC | 920±60d | 28.46±0.93d | 89.40±2.91d | 0.310±0.010b | 0.692±0.000de | 6270±273d | 8242±379c | 1128±85d |
|  | 0.10NBC | 771±56d | 21.07±1.57d | 66.20±4.92d | 0.273±0.000d | 0.452±0.034e | 5093±193e | 6407±579c | 932±107d |
|  | 0.05WBC | 1477±23b | 42.99±2.01c | 135.06±6.32c | 0.291±0.009c | 0.984±0.076c | 7989±152c | 13077±53c | 2267±24b |
|  | 0.10WBC | 1181±52c | 39.86±2.33c | 125.22±7.32c | 0.337±0.005a | 1.057±0.077c | 6906±267d | 11515±884c | 1667±139c |
|  | 0.05BCE | 2480±335a | 71.89±14.65b | 225.84±46.01b | 0.282±0.023cd | 1.659±0.438b | 9718±1079a | 19732±3887a | 3765±736a |
|  | 0.10BCE | 2557±77a | 82.32±2.71a | 258.62±8.52a | 0.322±0.001ab | 2.081±0.075a | 8975±128b | 21319±1552a | 3687±104a |

**Supplementary Table 4.** Changes in root distribution parameters of surface area, volume, tips of W7 and NP respectively under different biochar types in Experiment 1. Different letters in a single column indicate significant differences between treatments at *P*<0.05 (n=4).

| Cultivar | Treatment | Length (cm) | | Surface area (cm^2^) | | Projected area (cm^2^) | | Volume (cm^3^) | | Tips | |
| --- | --- | --- | --- | --- | --- | --- | --- | --- | --- | --- | --- |
|  |  | Primary | Secondary | Primary | Secondary | Primary | Secondary | Primary | Secondary | Primary | Secondary |
| W7 | CK | 278±4b | 630±16d | 41.84±0.78cd | 22.39±0.87d | 13.32±0.25cd | 7.13±0.28d | 0.577±0.030cd | 0.079±0.003d | 51±5c | 4080±190d |
|  | 0.05NBC | 301±1b | 647±7d | 46.50±0.45bc | 21.95±0.38d | 14.80±0.14bc | 6.99±0.12d | 0.666±0.015bc | 0.077±0.002de | 65±5c | 5425±96c |
|  | 0.10NBC | 214±58c | 426±126e | 33.43±9.05d | 16.07±5.05e | 10.64±2.88d | 5.11±1.61e | 0.480±0.125d | 0.058±0.019e | 120±50b | 4203±914d |
|  | 0.05WBC | 330±27b | 850±40bc | 53.44±3.34b | 30.88±0.21c | 17.01±1.06b | 9.83±0.07c | 0.822±0.022b | 0.110±0.001c | 188±67a | 7021±155a |
|  | 0.10WBC | 294±29b | 722±43cd | 49.77±3.68bc | 24.91±1.37d | 15.84±1.17bc | 7.93±0.44d | 0.805±0.046b | 0.087±0.005d | 72±1c | 6026±584bc |
|  | 0.05BCE | 447±69a | 1168±185a | 71.48±12.97a | 47.67±7.08a | 22.75±4.13a | 15.17±2.25a | 1.107±0.236a | 0.180±0.025a | 220±6a | 6808±1322ab |
|  | 0.10BCE | 406±8a | 959±51b | 65.58±3.66a | 39.82±2.82b | 20.87±1.17a | 12.67±0.90b | 1.016±0.102a | 0.152±0.013b | 129±17b | 5456±251c |
| NP | CK | 402±48c | 947±48b | 61.44±6.48c | 32.67±1.35b | 19.56±2.06c | 10.40±0.43b | 0.885±0.081c | 0.115±0.005b | 87±15cd | 6558±562c |
|  | 0.05NBC | 305±4cd | 606±55d | 49.17±0.51cd | 20.71±1.36cd | 15.65±0.16cd | 6.59±0.43cd | 0.754±0.029cd | 0.072±0.004cd | 84±7cd | 6183±279c |
|  | 0.10NBC | 255±13d | 513±43d | 36.42±1.83d | 18.33±1.93d | 11.59±0.58d | 5.84±0.61d | 0.476±0.022d | 0.066±0.007d | 79±23cd | 5014±171d |
|  | 0.05WBC | 408±13c | 1054±8b | 68.50±3.40c | 35.95±0.25b | 21.81±1.08c | 11.44±0.08b | 1.102±0.071c | 0.127±0.001b | 100±16c | 7888±135b |
|  | 0.10WBC | 386±16c | 772±33c | 63.43±3.71c | 26.08±1.44c | 20.19±1.18c | 8.30±0.46c | 1.014±0.079c | 0.093±0.006c | 60±2d | 6844±270c |
|  | 0.05BCE | 605±186b | 1697±258a | 96.95±32.40b | 63.74±9.75a | 30.86±10.31b | 20.29±3.10a | 1.531±0.585b | 0.231±0.036a | 156±35b | 9054±1439a |
|  | 0.10BCE | 773±58a | 1746±20a | 129.8±10.56a | 67.06±0.3a | 41.32±3.36a | 21.34±0.10a | 2.153±0.199a | 0.246±0.002a | 197±17a | 8769±113ab |

**Supplementary Table 5.** Rice shoot and root dry biomass per pot of W7 under 0%, 0.01%, 0.05% and 0.1% BCE dosage in Experiment 2a. Different letters in a single column indicate significant differences between treatments at *P*<0.05 (n=4).

| Biochar dose | Shoot biomass (g) | Root biomass (g) |
| --- | --- | --- |
| 0% | 2.28±0.14ab | 0.74±0.05b |
| 0.01% | 2.55±0.33ab | 0.90±0.04a |
| 0.05% | 2.58±0.14a | 0.95±0.08a |
| 0.10% | 2.22±0.16b | 0.78±0.06b |
| 0.10%+Water | 1.41±0.27 | 0.81±0.04 |

**Supplementary Table 6.** Changes in nutrients content of nitrogen, phosphorus and potassium by rice shoot and root, and nutrient transfer coefficient of the seedlings across the dosages of 0%, 0.01%, 0.05%, 0.10% BCE and 0.10% BCE alone in Experiment 2a. Different letters in a single column indicate significant differences between treatments at *P*<0.05 (n=4).

|  | Treatment | N | P | K |
| --- | --- | --- | --- | --- |
| Shoot (mg) | 0% | 88.62±4.29c | 13.36±0.51b | 55.53±3.41bc |
|  | 0.01% | 114.4±5.97a | 17.42±1.10a | 67.38±2.66a |
|  | 0.05% | 100.96±7.69b | 17.30±1.32a | 59.55±5.26b |
|  | 0.10% | 84.53±6.77c | 15.98±0.84a | 50.59±3.30c |
|  | 0.10%+Water | 29.13±8.53 | 2.57±0.52 | 31.87±7.48 |
| Root (mg) | 0% | 19.81±1.86b | 3.37±0.09b | 13.89±1.26b |
|  | 0.01% | 28.71±5.36a | 4.82±0.81a | 20.65±5.34a |
|  | 0.05% | 24.7±4.26ab | 4.3±0.92ab | 18.76±4.49ab |
|  | 0.10% | 19.32±2.79b | 3.44±0.18b | 13.63±1.29b |
|  | 0.10%+Water | 12.24±2.24 | 1.2±0.08 | 13±0.83 |
| Transfer coefficient  (%) | 0% | 22.33±1.28b | 25.27±0.89ab | 25.02±1.97b |
|  | 0.01% | 26.95±2.08a | 28.23±2.23a | 31.43±5.91ab |
|  | 0.05% | 24.34±2.46ab | 24.74±4.16ab | 31.22±5.12a |
|  | 0.10% | 22.93±3.32b | 21.53±0.93b | 26.99±2.53ab |
|  | 0.10%+Water | 43.87±11.98 | 46.32±12.86 | 43.61±9.24 |

**Supplementary Table 7.** Rice leaf gas exchange capacity (Transpiration rate, *E*; Net photosynthetic rate, *A*; Intercellular CO_2_ concentration, *Ci* and Stomatal conductance, *g_sw_*) of Wuyunjing 7 under 0%, 0.01% and 0.1% BCE dosage in Experiment 2b. The SPAD value in the youngest fully expanded leaves was estimated using a portable chlorophyll meter (SPAD-502, Soil-Plant Analysis Development Section, Konica Minolta Co. Ltd., Tokyo, Japan). Water use efficiency by rice was also calculated, including instantaneous water use efficiency (WUE, *A*/*E*) as net photosynthesis rate divided by transpiration rate and intrinsic water use efficiency (WUEi, *A*/*g_sw_*) as net photosynthesis rate divided by stomatal conductance at leaf scale (Seibt et al., 2008). Different letters in a single column indicate significant differences between treatments at *P*<0.05 (n=4).

| Biochar dose | SPAD | Area  (cm^2^) | *E*  (mmol m^-2^ s^-1^) | *A*  (µmol m^-2^ s^-1^) | *Ci*  (µmol mol^-1^) | *g_sw_*  (mol m^-2^ s^-1^) | WUE | WUEi |
| --- | --- | --- | --- | --- | --- | --- | --- | --- |
| 0% | 26.35±0.47c | 25.47±1.60b | 7.27±1.04a | 19.65±1.69b | 288.74±7.08a | 0.39±0.06b | 2.72±0.21b | 50.57±6.18b |
| 0.01% | 31.03±0.93a | 30.03±2.87a | 7.80±0.27a | 23.96±1.68a | 288.68±4.82a | 0.47±0.05a | 3.07±0.20b | 51.28±2.92b |
| 0.10% | 29.24±0.46b | 31.78±2.63a | 4.88±0.52b | 18.41±0.83b | 270.91±8.46b | 0.29±0.03c | 3.79±0.31a | 63.99±5.48a |

References:

Nakagawa, S., and Cuthill, I. (2007). Effect size, confidence interval and statistical significance: a practical guide for biologists. Biol. Rev. 82(4), 591-605. doi: 10.1111/j.1469-185X.2007.00027.x

Seibt, U., Rajabi, A., Griffiths, H., and Berry, J. (2008). Carbon isotopes and water use efficiency: sense and sensitivity. Oecologia 155(3), 441-454. doi: 10.1007/s00442-007-0932-7.

**Supplementary Table 8.** Rice root morphology of Wuyunjing 7 under 0%, 0.01%, 0.05% and 0.1% BCE dosage and 0.1% BCE alone in Experiment 2a. Different letters in a single column indicate significant differences between treatments at *P*<0.05 (n=4).

|  | Length  (cm) | Projected area  (cm^2^) | Surface area  (cm^2^) | Average diameter  (mm) | Volume  (cm^3^) | Tips | Forks | Crossings |
| --- | --- | --- | --- | --- | --- | --- | --- | --- |
| 0% | 1296±50c | 35.35±3.04b | 106.78±5.16b | 0.273±0.013ab | 0.713±0.054b | 4562±369a | 11144±957c | 3177±274c |
| 0.01% | 1914±70a | 55.56±2.74a | 174.55±8.59a | 0.282±0.015a | 1.248±0.128a | 5047±590a | 17203±627a | 5382±291a |
| 0.05% | 1742±134ab | 49.78±3.91a | 156.38±12.30a | 0.286±0.012a | 1.119±0.111a | 4598±562a | 16402±869ab | 4910±347a |
| 0.10% | 1565±172b | 39.38±6.61b | 123.71±20.76b | 0.256±0.016b | 0.782±0.180b | 4534±90a | 14772±1375b | 3986±588b |
| 0.10%+Water | 2753±96 | 65.46±2.82 | 205.64±8.86 | 0.237±0.004 | 1.25±0.056 | 11487±1134 | 22171±2252 | 6470±253 |

**Supplementary Table 9.** Rice root distribution of Wuyunjing 7 under 0%, 0.01%, 0.05% and 0.1% BCE dosage and 0.1% BCE alone in Experiment 2a. Different letters in a single column indicate significant differences between treatments at *P*<0.05 (n=4).

| Biochar dose | Length (cm) | | Surface area (cm^2^) | | Projected area (cm^2^) | | Volume (cm^3^) | | Tips | |
| --- | --- | --- | --- | --- | --- | --- | --- | --- | --- | --- |
|  | Primary | Secondary | Primary | Secondary | Primary | Secondary | Primary | Secondary | Primary | Secondary |
| 0% | 370±17b | 889±12c | 54.46±4.91b | 29.46±0.99c | 18.05±1.91b | 9.38±0.31c | 0.818±0.135b | 0.091±0.005c | 44±5a | 4152±312b |
| 0.01% | 512±26a | 1371±59a | 80.25±7.08a | 45.58±1.73a | 26.3±2.04a | 14.51±0.55a | 1.302±0.139a | 0.142±0.006a | 61±9a | 4895±524a |
| 0.05% | 477±5a | 1261±71a | 78.02±6.88a | 39.93±3.72b | 23.95±1.57a | 12.71±1.18b | 1.237±0.149a | 0.131±0.017ab | 57±13a | 4864±186a |
| 0.1% | 396±64b | 1130±117b | 63.51±13.96b | 37.6±3.78b | 18.51±3.76b | 12.15±1.08b | 0.822±0.199b | 0.116±0.012b | 57±13a | 4427±117ab |
| 0.1%+Water | 661±35 | 2069±69 | 97.67±0.8 | 67.43±2.52 | 30.38±1.43 | 21.46±0.80 | 1.348±0.035 | 0.213±0.004 | 105±12 | 11482±964 |

**Supplementary Table 10.** Changes in shoot and root water content of Wuyunjing 7 with different dosage BCE treatments in Experiment 2a and 2b. Different letters in a single column indicate significant differences between treatments at *P*<0.05 (n=4).

| Dosages | 2a | | 2b | |
| --- | --- | --- | --- | --- |
|  | Shoot (%) | Root (%) | Shoot (%) | Root (%) |
| 0% | 78.25±1.15a | 78.66±7.59b | 82.90±0.29a | 90.56±0.29b |
| 0.01% | 78.40±1.62a | 88.48±1.22a | 83.29±0.55a | 91.25±0.27a |
| 0.05% | 77.48±0.47a | 87.07±1.59a | / | / |
| 0.10% | 77.91±0.61a | 85.51±3.01a | 82.87±0.44a | 90.60±0.47b |

**Supplementary Table 11.** Putative identifications by gas chromatograph-mass spectrometer (GC-MS) of compounds in hot water extract from maize residue biochar.

| Retention Time | Compound | Y/N | Retention Time | Compound | Y/N |
| --- | --- | --- | --- | --- | --- |
| 9.141 | Trifluoroacetamide | Y | 29.278 | Nonanoic | Y |
| 9.654 | Ethane-1,2-diol (ethylene glycol) | — | 30.126 | Cinnamic acid | — |
| 10.011 | Acetamide | Y | 31.343 | 3Hydroxy-2,3-didehydrosebacic acid | — |
| 11.622 | 2-Butenoic acid | Y | 31.923 | Decanoic acid | Y |
| 11.930 | Silanamine | Y | 32.770 | 2-pentanoic acid | — |
| 12.510 | N-Trimethylsilymethoxyamine | Y | 33.116 | 1H-indole-2,3-dione | — |
| 14.629 | Butanoic acid | Y | 33.863 | 1,3-Bezoxazol-2-amine | — |
| 15.454 | Ethybis(trimethylsily)amine | Y | 36.339 | Tartaric acid | — |
| 15.766 | N, N-Diethy(trimethylsily)carbamate | — | 36.629 | Dodecanoic acid | Y |
| 10.034 | Propanoic acid | Y | 37.020 | Butylethylmalonic acid | — |
| 16.447 | Lactic acid | Y | 37.355 | 1,2-Benzenedicarboxylic acid | Y |
| 16.670 | Butane | — | 38.818 | Oxanilic acid | — |
| 17.964 | Acetic acid | Y | 40.849 | Tetradecanoic acid | Y |
| 18.912 | Ethanimidic acid | Y | 41.161 | Arabino-Hexonic acid | — |
| 19.313 | Ethanedioic acid | Y | 42.802 | n-Pentadecanoic acid | — |
| 19.693 | L-Aspartic acid | — | 43.739 | Glutaric acid | — |
| 20.429 | 4-cyclohexene-1,2-discarboxylic acid | — | 44.688 | Hexadecanoic acid | Y |
| 20.964 | Alanine | Y | 46.503 | 1,6-Anhydroglucose | Y |
| 21.410 | Benzenepropanoic acid | Y | 47.713 | Oleic acid | Y |
| 22.916 | Benzoic acid | — | 47.858 | Octadecanedioic acid | Y |
| 24.232 | Propanedioic acid | Y | 48.205 | Heptadecanoic acid | Y |
| 24.936 | Glycine | Y | 48.428 | 9,12-Octadecadiynoic acid | — |
| 25.494 | Benzoic acid trimethylsilyl ester | Y | 49.265 | Arachidonic acid | — |
| 26.319 | Octanoic acid | — | 53.709 | Camphoric acid | Y |
| 26.598 | Phosphoric acid | Y | 55.849 | Trehalose | Y |
| 26.788 | Glycerol | Y | 56.492 | 2-Dodecenedioic acid | — |
| 27.548 | 2-Butenedioic acid | Y | 10.588 | N, N-Dimethylglycine | Y |
| 27.927 | Butanedioic acid | Y | 19.774 | 1,2-Bistrimethylsiloxyethane | Y |
| 28.474 | Fumaric acid | Y | 20.900 | N, N-Diethyl-1,1,1-trimethylsilylamine | Y |
| 28.675 | Malonic acid | — | 31.332 | 2,4-1midazolidinedione | Y |
| 28.898 | Methylmaleic acid | — | 56.485 | Trans-Tranmatic acid | Y |

Note: Y means detected in the extract, — means undetected in the extract. Data of GC-MC was provided by YingMei Lou (Lou, 2015).

Lou, Y. (2015). Analysis of water extract from biochar and its application on vegetable growth as foliar spray. [master’s thesis]. [Nanjing (NJ)]: Nanjing Agricultural University

**Supplementary Table 12.** List of rice growth traits that were used to calculate relative changes by the Z-score normalization method under BCE dosages in Experiment 2a.

| Agronomic traits | Heigh |
| --- | --- |
|  | Fresh shoot/root biomass |
|  | Total fresh biomass |
|  | Dry shoot/root biomass |
|  | Total dry biomass |
|  | R/S ratio |
| Nutrients | Shoot N/P/K concentration |
|  | Root N/P/K concentration |
|  | Shoot N/P/K content |
|  | Root N/P/K content |
|  | N/P/K transfer coefficient |
| Root morphology | Depth |
|  | Total/Primary/ Secondary root length |
|  | Total/Primary/Secondary root projected area |
|  | Total/Primary/Secondary root surface area |
|  | Average diameter |
|  | Total/Primary/Secondary root volume |
|  | Total/Primary/Secondary root tips |
|  | Forks |
|  | Crossings |
